# Biomaterial-assisted local oxygenation safeguards the prostimulatory phenotype and functions of human dendritic cells in hypoxia

**DOI:** 10.3389/fimmu.2023.1278397

**Published:** 2023-12-07

**Authors:** Khushbu Bhatt, Alexandra Nukovic, Thibault Colombani, Sidi A. Bencherif

**Affiliations:** ^1^ Department of Pharmaceutical Sciences, Northeastern University, Boston, MA, United States; ^2^ Department of Chemical Engineering, Northeastern University, Boston, MA, United States; ^3^ Department of Bioengineering, Northeastern University, Boston, MA, United States; ^4^ Harvard John A. Paulson School of Engineering and Applied Sciences, Harvard University, Cambridge, MA, United States

**Keywords:** oxygen, hyaluronic acid, cryogel, hypoxia, dendritic cells, immunotherapy

## Abstract

Dendritic cells (DCs), professional antigen-presenting cells, function as sentinels of the immune system. DCs initiate and fine-tune adaptive immune responses by presenting antigenic peptides to B and T lymphocytes to mount an effective immune response against cancer and pathogens. However, hypoxia, a condition characterized by low oxygen (O_2_) tension in different tissues, significantly impacts DC functions, including antigen uptake, activation and maturation, migration, as well as T-cell priming and proliferation. In this study, we employed O_2_-releasing biomaterials (O_2_-cryogels) to study the effect of localized O_2_ supply on human DC phenotype and functions. Our results indicate that O_2_-cryogels effectively mitigate DC exposure to hypoxia under hypoxic conditions. Additionally, O_2_-cryogels counteract hypoxia-induced inhibition of antigen uptake and migratory activity in DCs through O_2_ release and hyaluronic acid (HA) mediated mechanisms. Furthermore, O_2_-cryogels preserve and restore DC maturation and co-stimulation markers, including HLA-DR, CD86, and CD40, along with the secretion of proinflammatory cytokines in hypoxic conditions. Finally, our findings demonstrate that the supplemental O_2_ released from the cryogels preserves DC-mediated T-cell priming, ultimately leading to the activation and proliferation of allogeneic CD3+ T cells. This work emphasizes the potential of local oxygenation as a powerful immunomodulatory agent to improve DC activation and functions in hypoxia, offering new approaches for cancer and infectious disease treatments.

## 1 Introduction

DCs play a critical role in the immune system, bridging the gap between the innate and adaptive immune responses. Their primary functions includes the detection and uptake of antigens, activation, and migration, followed by priming and proliferation of T cells (1). However, the effectiveness of DCs can be significantly impaired under hypoxic conditions (0.5–3% O_2_), which are commonly present in physiological tissues, solid tumors, and sites of inflammation (2, 3).

The hypoxic tumor microenvironment is particularly detrimental for DC functions. Hypoxia is a distinctive hallmark of solid tumors, arising from the rapid proliferation of cancer cells outpacing the growth of blood vessels supplying O_2_ (4). The hypoxic stress triggers the induction of transcription factors, such as hypoxia-inducible factors (HIFs) in inflamed and cancerous tissues, initiating a cascade of immunosuppressive effects on immune cells (5–10). Hypoxia has been reported to impair various aspects of DC functions, including antigen uptake, maturation, antigen presentation, and their ability to stimulate T-cell responses, thereby contributing to immune evasion by tumors (2, 3, 11). In particular, hypoxia inhibits the phagocytic ability of DCs to endocytose antigens, such as dextran, lipopolysaccharide (LPS), zymosan, and necrotic tumor cells (12–14). Additionally, hypoxia can downregulate the expression of major histocompatibility complex (MHC) class II molecules, such as HLA-DR, as well as co-stimulatory molecules, including CD40 and CD86, on DCs, thus impairing their ability to present antigens (12, 15–17). Moreover, hypoxia significantly decreases the migratory activity of DCs towards draining lymph nodes by suppressing matrix metalloproteinases and chemokine receptors such as CCR7, consequently impairing efficient T-cell priming (12, 15, 16, 18). Furthermore, hypoxia induces the expression of immune checkpoint molecules, such as PD-L1 on DCs, leading to the inhibition of T-cell function and overall suppression of anti-tumor immune responses (19).

Given the significant impact of hypoxia on DC functions and the consequent impairment of immune responses, there is a critical need to develop and test strategies to overcome hypoxia-induced immunosuppression of DCs. Such approaches would be useful in eliminating hypoxic conditions within the tumor microenvironment and local inflammation, thereby creating an immune-permissive microenvironment and ultimately restoring robust immune responses. Several approaches have been explored to eliminate hypoxia-induced immunosuppression, including supplemental oxygenation, HIF inhibitors, A2AR antagonists, and CD73 inhibitors (20–26). However, their utility is limited by off-target toxicities, systemic inflammatory changes, and low monotherapy efficacy (21, 23–26). Recently, innovative O_2_-generating biomaterials have emerged as a promising approach for modulating hypoxic microenvironments (27–36). Specifically, our lab has recently reported on injectable and macroporous O_2_-cryogels, composed of calcium peroxide (CaO_2_) particles and acrylate-PEG-catalase (APC), and fabricated with methacrylated hyaluronic acid (HAGM) via cryopolymerization (37–40). Upon hydrolysis, CaO_2_ releases O_2_ and hydrogen peroxide (H_2_O_2_) as a byproduct, which catalase degrades to produce O_2_ and water (H_2_O). Furthermore, O_2_-cryogels were demonstrated to restore T-cell-mediated cytotoxicity in hypoxic tumors *in vitro* and *in vivo* (37). Moreover, biomaterial implantation is commonly associated with hypoxia due to inevitable delay in the vascularization process (41–45). The inclusion of an O_2_-releasing construct is a highly desirable approach to enhance the viability and functions of immune cells. To that end, we have also reported on the co-adjuvant role of O_2_, released from O_2_-cryogels, in the context of improving the efficacy of a protein-based COVID-19 vaccine (42). However, despite acknowledging the potential of these O_2_-generating biomaterials, our understanding of their complete functionality remains limited. Gaining a deeper insight into the effects of localized oxygenation on immune cells is crucial before progressing toward clinical translation.

**Figure 1:**
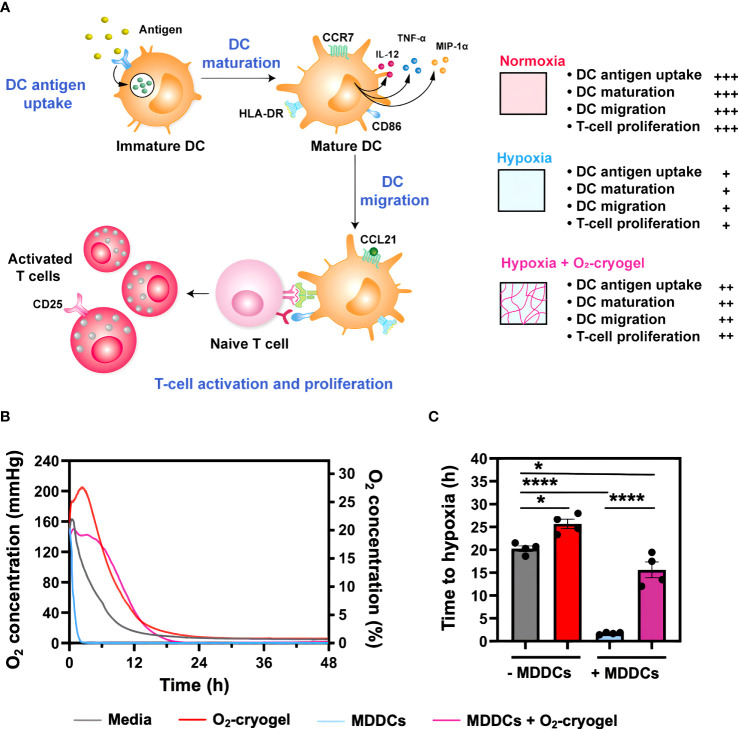
O_2_-cryogels mitigate the exposure of MDDCs to hypoxia. **(A)** Graphical abstract depicting local oxygenation from O_2_-cryogels preserves the proinflammatory function of DCs. Immature DCs identify and internalize pathogenic antigens via PRRs, initiating antigen processing and presentation via MHC molecules. This process is accompanied by DC maturation and upregulation of activation and co-stimulatory molecules, including CD40, CD86, and CCR7, as well as the secretion of proinflammatory cytokines and chemokines, such as IL-12p70, TNF-α, and MIP-1α. Following this, DCs migrate to draining lymph nodes in response to the chemokine gradient of CCL21, signaling through CCR7 receptor. Here, they prime naive T cells, triggering their activation and proliferation, and thereby mounting an effective immune response. However, the activity of DCs can be substantially compromised under hypoxic conditions found in physiological tissues, solid tumors, and sites of inflammation. Local O_2_ supply via O_2_-cryogels can significantly prevent hypoxia-induced inhibition of DC functions, including antigen uptake, maturation, migration, as well as T-cell activation and proliferation, and restore their proinflammatory activity. **(B)** Profile of O_2_ concentration measured in media with and without MDDCs and O_2_-cryogels. MDDCs (1.5x10^5^ cells/well) were cultured under hypoxic conditions (1% O_2_) for 48 h in the presence and absence of O_2_-cryogels, and O_2_ tension in the media was monitored using contactless sensor spots. Controls included medium alone, or cells cultured in medium in the presence and absence of O_2_-cryogels. Data are representative of three independent experiments and presented as mean of n = 4 replicates. **(C)** Time it takes for the MDDCs to become hypoxic in cell culture media in the presence and absence of O_2_-cryogels. Data are representative of three independent experiments and presented as mean ± SD of n = 4 replicates. Statistical analysis was performed using one-way ANOVA and Tukey’s *post hoc* test using GraphPad software; *P < 0.05, ****P < 0.0001.

In this study, our focus was on investigating whether O_2_-cryogels can counteract the hypoxia-induced inhibition of key functions of human DCs, including antigen uptake, activation and maturation, secretion of proinflammatory cytokines, chemotaxis, as well as T-cell priming and proliferation (**Figure 1A**). To this end, we initially evaluated the capacity of O_2_-cryogels to reduce the duration of hypoxia exposure experienced by DCs under hypoxic conditions. Subsequently, we explored how O_2_-cryogels could mitigate the suppressive effects of hypoxia on the antigen uptake function of DCs. Additionally, we assessed the migratory behavior of oxygenated DCs under hypoxic conditions. Moreover, our study delved into the role of O_2_-cryogels in preventing the downregulation and restoring of maturation markers on hypoxic DCs following LPS stimulation. We also evaluated the ability of O_2_-cryogels to maintain the secretion of proinflammatory cytokines and chemokines by activated DCs in hypoxia. Finally, we examined the capability of O_2_-cryogels to preserve DC-mediated activation and proliferation of allogenic T cells in a hypoxic environment. Overall, the outcomes of this study would provide valuable insights into the development of effective immunotherapies utilizing oxygenation, particularly for conditions where hypoxia is prevalent, such as cancer and inflammatory disorders.

## 2 Methods

### 2.1 Generation of human monocyte-derived dendritic cells and culture conditions

Human MDDCs were generated as previously described (46). Briefly, MDDCs were differentiated from cryopreserved human CD14+ monocytes, isolated using a negative selection technique (Stemcell Technologies). Monocytes were cultured in ImmunoCult™-ACF Dendritic Cell Medium (Stemcell Technologies) supplemented with 50 ng/mL recombinant human granulocyte-macrophage colony-stimulating factor (GM-CSF) (R&D systems) and 50 ng/mL recombinant human interleukin-4 (IL-4) (R&D systems) for 7 days. On day 1, monocytes were plated in 6-well plates (1x10^6^ cells/mL, 3 mL/well). Then, half of the media was replaced with fresh, complete media on days 3,5 and 7. On Day 7, MDDCs were ready to use for downstream applications.

### 2.2 Normoxic and hypoxic culture conditions

To maintain normoxic conditions, MDDCs were incubated at 37°C in a humidified incubator (5% CO_2_ and 95% air). To simulate hypoxic conditions, cells were incubated in a humidified tri-gas incubator (Heracell VIOS 160i, Thermo Fisher Scientific) flushed with a mixture of 1% O_2_, 5% CO_2,_ and 94% N_2_.

### 2.3 Fabrication of cryogels

O_2_-cryogels were fabricated by free-radical cryopolymerization as previously described (37). Briefly, HA (Sigma Aldrich) was methacrylated using glycidyl methacrylate (GM) (Sigma Aldrich) to provide the functional groups required for free-radical cross-linking (47). To degrade H_2_O_2_, catalase was chemically coupled to acrylate-PEG-NHS (3:1 molar ratio) to make O_2_-cryogels.

To fabricate O_2_-cryogels, HAGM (4% w/v) was combined with APC (2% w/v) and CaO_2_ particles (1% w/v) in deionized H_2_O (1 mL). Tetramethyl ethylenediamine (TEMED) (0.56% w/v) (Sigma Aldrich) and ammonium persulfate (APS) (1.12% w/v) (Sigma Aldrich), the free-radical catalyst and initiator system, were added to the precooled polymer mixture at 4°C. The solution was immediately poured into pre-cooled Teflon molds (4°C) and transferred to a −20°C freezer for 16 h. CaO_2_-containing HAGM cryogels (i.e., O_2_-cryogels) were fabricated by redox-induced free radical cryopolymerization process at −20°C. Then, O_2_-cryogels were thawed at room temperature (RT) to remove ice crystals, sanitized (70% ethanol, 10 min), and subsequently washed 5 times with phosphate-buffered saline (PBS) before utilization. HAGM cryogels (CaO_2_-free cryogel), which do not generate O_2_, were used as blank control cryogels in our experiments. This allowed us to distinguish the effect of O_2_ when comparing results with O_2_-cryogels. The latter primarily consist of HAGM cryogels embedded with O_2_-releasing CaO_2_ particles. Additionally, PEG cryogels (HA-free cryogels) were used to investigate the influence of HA, a biopolymer known for its intrinsic biological properties that could potentially influence DC behavior. Given that these PEG cryogels lack HA, any disparities observed between the PEG cryogels and HA-containing cryogels (HAGM and O_2_-cryogel) could be attributed to the presence of HA. To fabricate PEG cryogels, polyethylene glycol methacrylate (PEGDM) was synthesized as previously described (48, 49). PEGDM (10% w/v) was dissolved in H_2_O, and PEG cryogels were fabricated similarly to the above-mentioned method.

### 2.4 Oxygen concentration measurement

The partial pressure of O_2_ (torr or mmHg) in cell culture media was measured using optical sensor spots (OXSP5-ADH - Pyroscience). Sensors were adhered onto the bottom of 96-well plates using silicone glue (SPGLUE, PyroScience) and dried for 16 h. Measurements were conducted in tri-gas cell culture incubators under normoxic (18.6% O_2_) and hypoxic (1% O_2_) conditions. To measure paracellular O_2_, sensors were submerged in 200 µL of cell culture media containing 1.5x10^5^ MDDCs in the presence or absence of O_2_-cryogels. Media alone was used as control.

### 2.5 Flow cytometry analysis

Cells were washed in PBS (Gibco) and stained with fixable viability dye (ThermoFisher Scientific) for 15 min at 4°C. Then, Fc receptors on cells were blocked using Human TruStain FcX™ (Fc Receptor Blocking Solution, BioLegend) in FACS buffer (PBS + 2% FBS) for 10 min at 4°C and subsequently stained for surface markers at 4°C for 30 min using the following fluorescently-labeled antibodies diluted in FACS buffer: anti-human CD14 Antibody (clone M5E2, BioLegend), anti-human CD11c Antibody (clone 3.9, BioLegend), anti-human HLA-DR Antibody (clone L243, BioLegend), anti-human CD206 (MMR) Antibody (clone 15-2, BioLegend), anti-human CD40 Antibody (clone 5C3, BioLegend), anti-human CD80 Antibody (clone 2D10, BioLegend), anti-human CD83 Antibody (clone HB15e, BioLegend) anti-human CD86 Antibody (clone FUN-1, BD Biosciences), anti-human CD197 (CCR7) Antibody (clone G043H7, BioLegend), anti-human CD184 (CXCR4) Antibody (clone12G5, BioLegend), anti-human CD3 Antibody (clone UCHT1, BioLegend), anti-human CD4 Antibody (clone SK3, BioLegend), anti-human CD69 Antibody (clone FN50, BioLegend), anti-human CD25 Antibody (clone M-A251, BioLegend). Finally, the cells were fixed with IC fixation buffer (ThermoFisher Scientific) at 4°C for 30 min or overnight, followed by 3 washes with FACS buffer. Flow cytometry data were recorded on Attune NxT flow cytometer (Thermo Fisher Scientific), where at least 100,000 cells per sample were acquired and analyzed using FlowJo software (FlowJo LLC). Cell debris were excluded using forward scatter (FSC) and side scatter (SSC) properties, followed by gating single cells using area (A) and height (H) parameters (**Supplementary Figure 1B**). Subsequently, dead cells were removed using viability staining and MDDCs were defined as CD11c+ HLA-DR+.

### 2.6 Antigen uptake assay

pHrodo Green Dextran (ThermoFisher Scientific) and pHrodo Green-labeled ovalbumin (OVA) (ThermoFisher Scientific) were used as model antigens to test antigen uptake by MDDCs. Approximately 1.5×10^5^ cells MDDCs were preconditioned at 37°C in either normoxic or hypoxic conditions and at 4°C (negative control) for 24 h in the presence and absence of O_2_-cryogels (1 gel/well). Then, MDDCs were treated with media in the absence (negative control) or presence of the model antigens (pHrodo Green Dextran at 50 ug/mL or pHrodo Green-labeled OVA at 25 ug/mL) for 1.5 h. Antigen uptake was stopped by transferring the plates on ice and adding ice-cold PBS supplemented with 2% FBS. Next, cells were stained for viability and cell surface marker staining as described above. Antigen uptake capacity of the DCs was assessed using flow cytometry by calculating the mean fluorescence intensity (MFI) in the FITC channel.

### 2.7 Migration assay

MDDC migration was measured using a 96-well transwell system (CytoSelect™ 96-Well Cell Migration Assay, 5 µm pore size, Fluorometric Format, Cell Biolabs). MDDCs were preconditioned at 37°C in either normoxic or hypoxic conditions for 24 h in the presence and absence of O_2_-cryogels. After 24 h, gels were removed from the wells, the cells were harvested, and a total of 1.5×10^5^ MDDCs were added in the upper chamber of the transwell. The lower wells were filled with 150 µL media containing 1000 ng/mL recombinant human CCL21 (R&D systems). After 3 h of incubation under normoxic and hypoxic conditions, the migrated cells were harvested from the lower chamber and lysed using 4X Lysis Buffer/Cyquant® GR Dye according to the manufacturer’s protocol. The fluorescence was determined using a fluorescence plate reader at 480 nm/520 nm (Cytation 3, BioTek). A standard curve was constructed using a known number of MDDCs cultured under hypoxic and normoxic conditions (**Supplementary Figure 1D**). Number of migrated cells was determined by extrapolating the fluorescence readout from the standard curve generated using a known number of MDDCs in GraphPad software.

### 2.8 Activation assay

MDDCs were preconditioned in either hypoxic or normoxic conditions in the presence and absence of O_2_-cryogels for 24 h. For MDDC activation, cells were stimulated with 2 µg/mL LPS (*Invivo*gen) and 50 ng/mL of recombinant human IFN-γ protein (R&D systems) or 50 µg/mL Poly (I:C) (*Invivo*gen). After 24 h, the gels were removed, supernatants were collected, and cells were stained for viability and cell surface staining for DC activation markers as described above. DCs were imaged for their morphological differences using brightfield microscopy. Moreover, DC maturation markers were analyzed using flow cytometry by calculating the percent and mean fluorescence intensity (MFI) of the markers in their respective channels. Additionally, levels of proinflammatory cytokines and chemokines secreted by activated DCs were determined in the supernatant using Cytokine & Chemokine 34-Plex Human ProcartaPlex™ Panel 1A (ThermoFisher Scientific) and FLEXMAP 3D® System (Luminex).

In a different experimental setup, MDDCs were stimulated with LPS (2 µg/mL) and IFN-γ (50 ng/mL) and subjected to the following experimental conditions: (*i*) normoxia for 48 h, (*ii*) hypoxia for 48 h, (*iii*) preconditioning in hypoxia for 6 h, followed by addition of O_2_-cryogels and further incubation in hypoxic conditions for an additional 42 h, and (*iv*) preconditioning in hypoxia for 6 h, followed by addition of O_2_-cryogels and incubation in hypoxic conditions for 18 h, after which they were transferred to normoxic conditions for an additional 24 h. After 48 h, the gels were removed and cells were stained for viability and cell surface staining for DC activation markers as described above. The ability of O_2_-cryogels to restore activation and maturation markers on hypoxic MDDCs was evaluated and compared with normoxic MDDCs. Additionally, a comparison was conducted between normoxic MDDCs and hypoxic MDDCs treated with O_2_-cryogels, which were subsequently transferred to normoxic conditions.

### 2.9 Mixed lymphocyte reaction assay

Activated MDDCs were treated with 50 µg/mL Mitomycin C (Sigma), washed 3 times with media and resuspended in ImmunoCult™-XF T Cell Expansion Medium (Stemcell Technologies). Human Peripheral Blood Pan-T Cells from an independent donor (Stemcell Technologies) were labeled with CellTrace™ Violet (CTV) Cell Proliferation dye (ThermoFisher Scientific) according to the manufacturer’s instructions. MDDCs (1.5×10^4^ cells/well) were co-incubated with CTV-labeled pan-T cells (1.5×10^5^) at a 1:10 ratio in the absence and presence of O_2_-cryogels for 6 days. Additionally, CTV-labeled T cells were stimulated with anti-CD3/anti-CD28 Dynabeads (aCD3/aCD28) (ThermoFisher Scientific) and cell stimulation cocktail of phorbol 12-myristate 13-acetate (PMA) and ionomycin (PMA-ionomycin) (ThermoFisher Scientific) as positive controls for activation and proliferation. Then, cells were stained for viability, cell surface lineage and activation markers for T cells such as CD3, CD4, CD8, and CD25 as previously described. Allogenic T-cell proliferation was assessed by tracking CTV dilution among CD3+ T-cell population. Supernatants were collected and secretion of Th1 cytokines such as IFN-γ, IL-2, TNF-α and TNF-β were analyzed using Cytokine & Chemokine 34-Plex Human ProcartaPlex™ Panel 1A (ThermoFisher Scientific) and FLEXMAP 3D® System (Luminex).

### 2.10 Statistical analysis

Data are representative of three independent experiments with three individual donors and n=3*–*5 replicates in each experiment. Data were analyzed using GraphPad Prism 9 software (GraphPad) and presented as mean ± SD. Statistical analysis was performed using 2-way ANOVA and Tukey’s *post hoc* test using GraphPad software; *P<0.05, **P < 0.01, ***P < 0.001 ****P < 0.0001.

## 3 Results

### 3.1 O_2_-cryogels prevent MDDCs from experiencing sustained hypoxia

Upon infiltration in cold tumors, DCs are exposed to hypoxia, which inhibits their innate immune function, ultimately impairing their ability to initiate adaptive immune responses effectively. Therefore, we investigated the capacity of O_2_-cryogels to shield MDDCs from exposure to hypoxia and subsequently prevent the inhibitory effects induced by hypoxia. MDDCs (1.5x-10^5^ cells/well) were cultured under normoxic (18.6% O_2_) (**Supplementary Figure 1A**) and hypoxic conditions (1% O_2_) for 48 h, both in the presence and absence of O_2_-cryogels. The O_2_ tension in the media was monitored using contactless sensor spots (**Figures 1B, C**). Controls included medium alone, or cells cultured in medium in the presence or absence of cryogels. As expected, MDDCs cultured in hypoxia reach 1% O_2_ level within 2 h, compared to the 20 h required for the medium alone, highlighting the cellular O_2_ consumption rate. Strikingly, O_2_-cryogels released O_2_ for 16 h under hypoxic conditions and postponed cellular hypoxia by 13 h. In addition, MDDCs cultured with O_2_-cryogels maintained a steady-state of 1% O_2_, suggesting that these biomaterials continued to release O_2_ for an extended duration, covering the cellular consumption of O_2_ by MDDCs. Collectively, these results indicate that O_2_-cryogels can prevent MDDCs from experiencing hypoxia by delaying the time it takes for the cells to become hypoxic.

### 3.2 O_2_-cryogels mitigate hypoxia-induced inhibition of antigen uptake by MDDCs

**Figure 2:**
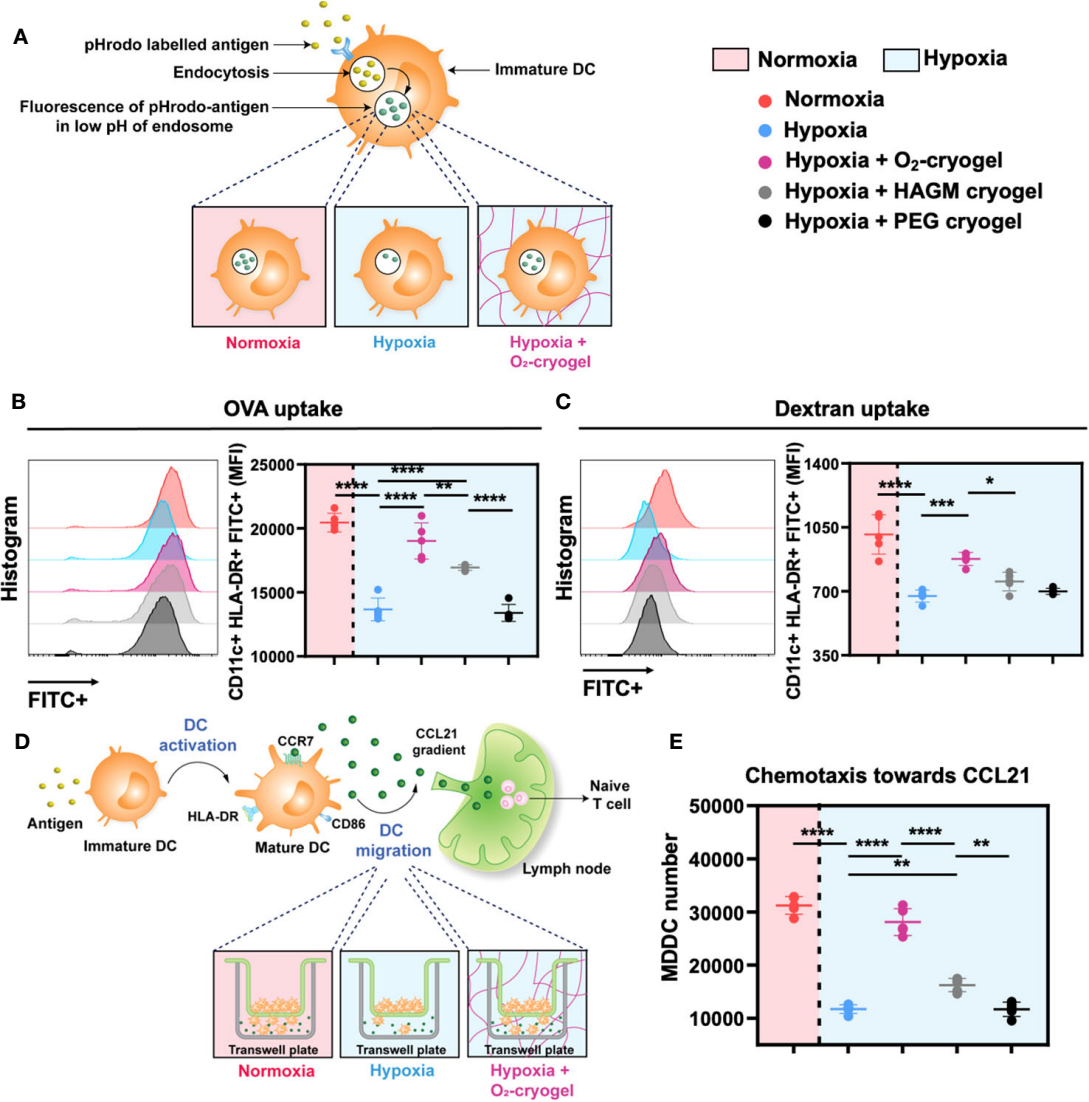
O_2_-cryogels preserve antigen uptake and chemotaxis function of MDDCs in hypoxia via O_2_ and HA-related mechanisms. **(A)** Schematic of antigen uptake by DCs occurring optimally in normoxia vs. inhibited in hypoxia *vs.* protected by O_2_-cryogels in hypoxia. MDDCs were preconditioned in either normoxia or hypoxia in cryogel-free medium or medium containing various cryogels (O_2_-cryogel, HAGM cryogel, PEG cryogel) for 24 (h) Then, the cells were exposed to pHrodo Green-labeled OVA and dextran for 1 h, washed, stained for surface markers and MFI in the FITC channel was analyzed using flow cytometry. pHrodo Green exhibits fluorescence in the low pH environment of the lysosome, serving as an indicator of phagocytosis. **(B)** OVA uptake by MDDCs preconditioned with various cryogels in normoxic and hypoxic conditions. **(C)** Dextran uptake by MDDCs preconditioned with various cryogels in normoxic and hypoxic conditions. **(D)** Schematic of migration of DCs occurring optimally in normoxia *vs.* inhibited in hypoxia *vs.* maintained by O_2_-cryogels in hypoxia. MDDCs were preconditioned in either normoxia or hypoxia in cryogel-free medium or medium containing various cryogels (O_2_-cryogel, HAGM cryogel, PEG cryogel) for 24h. Then, a transwell-based system was employed to assess the migratory activity of MDDCs. Medium-containing chemokine CCL21 was added in the lower chamber, whereas the preconditioned cells were added on the top chamber. Chemotaxis was allowed to take place for 3 h in hypoxic and normoxic conditions. Subsequently, the migrated cells in the lower chamber were lysed using Lysis Buffer/Cyquant® GR Dye, and the number of migrated cells was measured using fluorescence-based readout. **(E)** Chemotaxis of MDDCs induced by CCL21 in normoxic and hypoxic conditions post preconditioning with various cryogel formulations. Data are representative of three independent experiments and presented as mean ± SD of n = 4–5 replicates. Statistical analysis was performed using one-way ANOVA and Tukey’s *post hoc* test using GraphPad software; *P < 0.05, **P < 0.01, ***P < 0.001, ****P < 0.0001.

Immature DCs are known for their ability to detect and capture antigens, initiating both innate and adaptive immunity (50). However, this function is compromised under hypoxic conditions, resulting in a diminished innate immune response (13, 14). Therefore, we evaluated the endocytic ability of immature DCs to uptake the OVA model antigen and dextran in both normoxic and hypoxic conditions in the presence of O_2_-cryogels (**Figure 2A**). First, MDDCs were preconditioned in either normoxia or hypoxia in cryogel-free medium or medium containing various cryogels (O_2_-cryogel, HAGM cryogel, PEG cryogel) for 24 h. HAGM cryogel (CaO_2_-free cryogel) served as a control to determine the impact of O_2_ on the endocytic capacity of DCs, while PEG cryogel (HA-free cryogel) was used to investigate the influence of HA. Then, cells were exposed to OVA and dextran for 1 h, washed and stained for surface markers, and the Mean Fluorescence Intensity (MFI) was analyzed using flow cytometry. Antigen uptake at 4°C served as a negative control (**Supplementary Figure 1C**). Notably, OVA and dextran uptake by hypoxic MDDCs was markedly impaired in comparison to normoxia (13699 ± 887 vs 20453 ± 731 for OVA, 674 ± 33 *vs.* 1011 ± 108 for dextran). As expected, O_2_-cryogels effectively countered hypoxia-induced inhibition of antigen uptake, retaining up to 93% (19022 ± 1411 for OVA, 877 ± 36 for dextran) of the endocytic capacity (**Figures 2B, C**). Interestingly, HAGM cryogel also enhanced the antigen uptake ability of MDDCs in hypoxia (16939 ± 221 for OVA, 754 ± 51 for dextran), surpassing the effects of PEG cryogel (13369 ± 661 for OVA, 699 ± 17 for dextran). Taken together, our data suggest that O_2_-cryogels can preserve the antigen uptake function of MDDCs in hypoxic conditions, with this effect synergistically amplified by HA-mediated mechanisms.

### 3.3 O_2_-cryogels prevent hypoxia-induced inhibition of MDDC chemotaxis

Upon maturation, DCs migrate from inflamed tissues and travel through the lymphatics to the lymph nodes using CCL21-mediated chemotaxis (51). However, hypoxic conditions alter the migratory capacity of DCs towards these chemokine gradients, hampering their ability to reach lymph nodes to initiate and orchestrate a robust adaptive immune response (16). Therefore, we next evaluated the effect of O_2_-cryogels on CCL21-mediated migration of hypoxic DCs using a transwell-based system (**Figure 2D**). First, MDDCs were preconditioned in either normoxia or hypoxia using cryogel-free medium or medium containing various cryogels (O_2_-cryogel, HAGM cryogel, PEG cryogel) for 24 h. Subsequently, a medium containing the chemokine CCL21 was added in the lower chamber, and preconditioned cells were placed in the upper chamber. Chemotaxis was allowed to occur for 3 h under hypoxic and normoxic conditions. Afterward, the migrated cells in the lower chamber were lysed using Lysis Buffer/Cyquant^®^ GR Dye, and the number of migrated cells was quantified using fluorescence-based measurements. Notably, hypoxia substantially decreased the chemotactic ability of MDDCs towards CCL21 in comparison to normoxia (11722 ± 829 *vs.* 31249 ± 1653), a reduction that was alleviated by up to 90% using O_2_-cryogels (28111 ± 2531) (**Figure 2E**). Furthermore, HAGM cryogel also improved the chemotactic response compared to PEG cryogel (16243 ± 1257 v*s.* 11683 ± 1349). Overall, our data indicates that O_2_-cryogels can preserve the chemotactic response of MDDCs towards CCL21 in hypoxia by increasing O_2_ tension and providing HA-associated interactions.

### 3.4 O_2_-cryogels prevent hypoxia-induced inhibition of MDDC maturation

**Figure 3:**
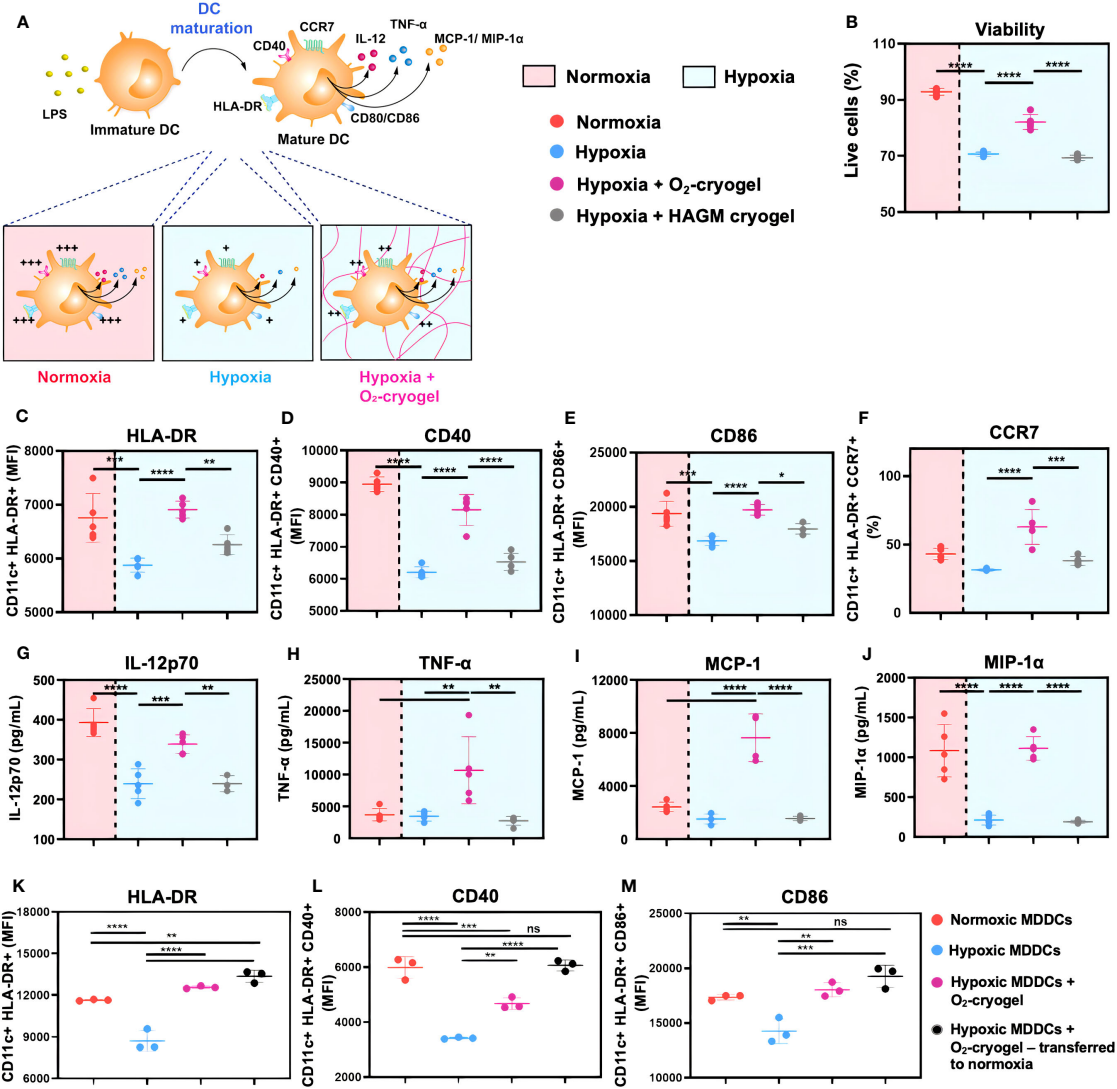
O_2_-cryogels safeguard the maturation of MDDCs in hypoxia. **(A)** Schematic of DCs optimally activated in normoxia *vs.* inhibited in hypoxia *vs.* rescued by O_2_-cryogels in hypoxia upon stimulation with LPS. **(B-J)** MDDCs were preconditioned in either normoxia or hypoxia in cryogel-free medium or medium containing various cryogels (O_2_-cryogel, HAGM cryogel) for 24 h in the presence of 2 µg/mL LPS and 50 ng/mL of IFN-γ. **(B)** Fraction of live MDDCs, MFI of **(C)** CD11c+ HLA-DR+, **(D)** CD11c+ HLA-DR+ CD40+, **(E)** CD11c+ HLA-DR+ CD86+ MDDCs, **(F)** Fraction of CD11c+ HLA-DR+ CCR7+ MDDCs, secretion of **(G)** IL-12p70, **(H)** TNF-α, **(I)** MCP-1, and **(J)** MIP-1α, 24 h after culture with different experimental conditions. **(K-M)** MDDCs were stimulated with 2 µg/mL LPS and 50 ng/mL of IFN-γ and subjected to different experimental conditions, *(i)* normoxia for 48 h, (*ii*) hypoxia for 48 h, (*iii*) preconditioned in hypoxia for 6 h, followed by addition of O_2_-cryogel and incubation in hypoxic conditions for another 42 h, (*iv*) preconditioned in hypoxia for 6 h, followed by addition of O_2_-cryogel and incubation in hypoxic conditions for 18 h and subsequently transferred to normoxia for another 24h. MFI of **(K)** CD11c+ HLA-DR+, **(L)** CD11c+ HLA-DR+ CD40+, and **(M)** CD11c+ HLA-DR+ CD86+ MDDCs subjected to various experimental conditions. Data are representative of three independent experiments and presented as mean ± SD of n = 4–5 replicates. Statistical analysis was performed using one-way ANOVA and Tukey’s *post hoc* test using GraphPad software; *P < 0.05, **P < 0.01, ***P < 0.001, ****P < 0.0001, ns, not significant.

DCs undergo activation and phenotypic maturation in response to various pattern recognition receptors (PRR) present on pathogens, including bacterial cell wall components, such as LPS, and molecular patterns associated with viral infections, such as Poly (I:C) (52). However, hypoxic conditions alter the maturation of DCs, which in turn may affect their ability to prime T-cell responses (16, 17). Therefore, we explored the potential of O_2_-cryogels to maintain and restore the phenotypic maturation of DCs in hypoxia upon activation with LPS + IFN-γ or Poly (I:C). First, MDDCs were preconditioned in either normoxia or hypoxia using cryogel-free medium or medium containing various cryogels (O_2_-cryogel, HAGM cryogel) for 24 h in the presence of 2 µg/mL LPS and 50 ng/mL of IFN-γ or 50 µg/mL Poly (I:C) (**Figures 3A–J**, **Supplementary Figures 2A–I**, **3A–E**, **4A–J**). Next, MDDCs were imaged using brightfield microscopy to inspect potential morphological differences (**Supplementary Figures 2A–D**). The cells were also stained for viability, and DC maturation markers were assessed by flow cytometry. No significant morphological differences were noted in activated MDDCs under various conditions. The cells displayed a typical DC morphology characterized by semi-adherent properties and protruding spikes (**Supplementary Figures 2A–D**). Notably, hypoxic DCs were smaller in size compared to normoxic DCs (111987 ± 1742 *vs*. 151411 ± 4565) as indicated by FSC-A, a parameter used to evaluate cell size by flow cytometry. Moreover, O_2_-cryogel-treated DCs retained 90% of their size compared to those in normoxia (135936 ± 3581) (**Supplementary Figures 2E–I**). Upon activation with LPS + IFN-γ or Poly (I:C) in hypoxia, the viability of MDDCs was significantly reduced compared to normoxic conditions (71 ± 1% *vs.* 93 ± 1%) (**Figure 3B**, **Supplementary Figures 3A**, **4A**). Remarkably, O_2_-cryogels protected the viability of MDDCs from the impact of hypoxia (82 ± 3%). Additionally, hypoxia downregulated the expression of HLA-DR, CD40, CD86 (in terms of MFI), and CCR7 (in terms of percent positive cells), compared to normoxia (**Figures 3C–F**, **Table 1**, **Supplementary Figures 3B–E, 4B–F, 4H–J**). As anticipated, O_2_-cryogels also shielded DCs from the inhibitory effects of hypoxia by maintaining 80–100% of their maturation markers (102% for HLA-DR, 91% for CD40, 102% for CD86, 145% for CCR7) compared to normoxia. Furthermore, hypoxia also led to a reduction in the secretion of proinflammatory cytokines and chemokines, such as Interleukin-12 (IL-12p70), Tumor necrosis factor alpha (TNF-α), Monocyte Chemoattractant Protein-1 (MCP-1), Macrophage Inflammatory Protein-1 alpha (MIP-1α) by MDDCs in hypoxia compared to normoxia (**Figures 3G–J**, **Table 1**, **Supplementary Figure 4G**). Additionally, O_2_-cryogels preserved and enhanced the capability of MDDCs to secrete these proinflammatory cytokines and chemokines (86% for IL-12p70, 290% for TNF-α, 314% for MCP-1, 103% for MIP-1α) compared to normoxia. Furthermore, the restorative effects of O_2_-cryogels on activated DCs previously exposed to hypoxia were evaluated and compared to normoxic DCs. (**Figures 3K–M**, **Supplementary Figures 3F–H**, **Table 2**). Strikingly, O_2_-cryogel-treated hypoxic DCs restored the levels of maturation and stimulation markers such as HLA-DR (by 108%), CD40 (by 78%), and CD86 (by 104%) compared to normoxia. Moreover, normoxia-conditioned MDDCs were compared to hypoxic MDDCs treated with O_2_-cryogels that were subsequently transferred to normoxic conditions. As expected, O_2_-cryogel-treated hypoxic DCs that were transferred to normoxia exhibited similar (CD40, CD86) or slightly higher (HLA-DR) levels of maturation markers. Altogether, our set of data strongly implies that O_2_-cryogels can not only preserve but also restore the phenotypic maturation of DCs, which is typically compromised in hypoxic conditions.

**Table 1:** Expression of cell surface markers and secretion of proinflammatory cytokines by LPS-activated MDDCs.

Markers	Normoxia	Hypoxia	Hypoxia + O_2_-cryogel
**HLA-DR (MFI)**	6754 ± 451	5876 ± 132	6910 ± 157
**CD40 (MFI)**	8952 ± 227	6207 ± 172	8152 ± 480
**CD86 (MFI)**	19365 ± 1145	16860 ± 433	19719 ± 485
**CCR7 (%)**	43 ± 4	31 ± 1	63 ± 13
**IL-12p70 (pg/mL)**	393 ± 35	239 ± 37	339 ± 24
**TNF-α (pg/mL)**	3681 ± 973	3454 ± 783	10662 ± 5262
**MCP-1 (pg/mL)**	2429 ± 360	1510 ± 373	7633 ± 1799
**MIP-1α (pg/mL)**	1084 ± 330	212 ± 62	1112 ± 148

**Figure 4:**
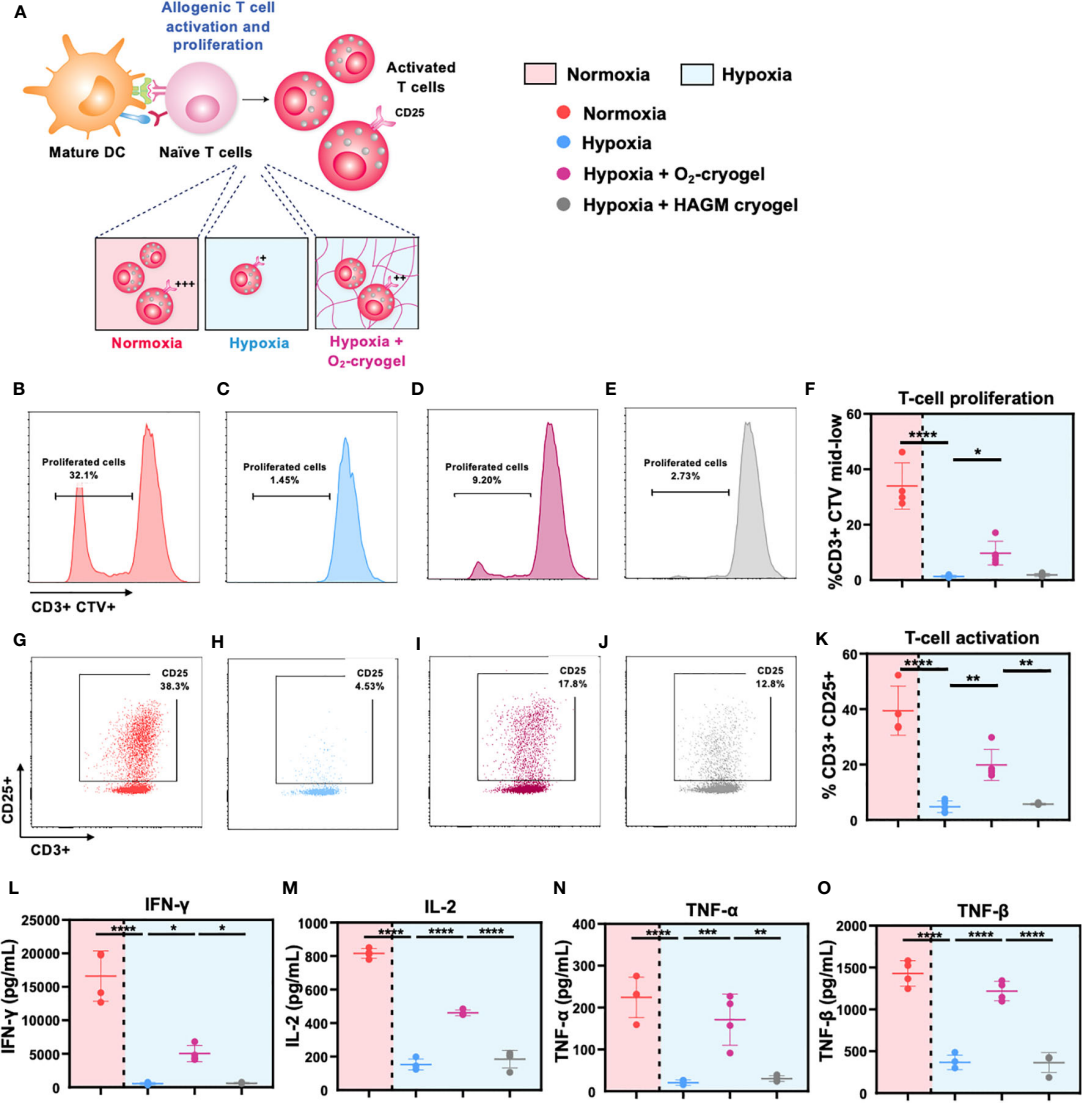
O_2_-cryogels maintain DC-mediated T-cell activation and proliferation under hypoxic conditions. **(A)** Schematic representation of DCs effectively priming naive T cells under normoxic conditions, resulting in their activation and proliferation. Conversely, hypoxic DCs fail to initiate T-cell activation and proliferation. However, local oxygenation provided by O_2_-cryogels under hypoxic conditions can partially preserve T-cell activation and proliferation. MDDCs were stimulated with LPS+IFN-γ preconditioned in either normoxia or hypoxia in cryogel-free medium or medium containing various cryogels (O_2_-cryogel, HAGM cryogel) for 48h. Next, activated MDDCs were co-cultured with CTV-labeled T cells from an independent donor in a 1:10 ratio for 6 days in cryogel-free medium or medium containing various cryogels (O_2_-cryogel, HAGM cryogel). Subsequently, cells were stained for T-cell lineage and activation markers and proliferation was assessed by monitoring CTV dilution using flow cytometry. Cytokine levels were assessed in the supernatant using Luminex. Fractions of **(B-F)** CD3+ CTV mid-low population, **(G-K)** CD3+ CD25+ cells, secretion of **(L)** IFN-γ, **(M)** IL-12p70, **(N)** TNF-α, and **(O)** TNF-β, 6 days after culture under different experimental conditions. Data are representative of three independent experiments and presented as mean ± SD of n = 4–5 replicates. Statistical analysis was performed using one-way ANOVA and Tukey’s *post hoc* test using GraphPad software; *P < 0.05, **P < 0.01, ***P < 0.001, ****P < 0.0001.

**Table 2:** Expression of cell surface markers by LPS-activated MDDCs under various conditions.

Marker (MFI)	Normoxic MDDCs	Hypoxic MDDCs	Hypoxic MDDCs + O_2_-cryogel	Hypoxic MDDCs + O_2_-cryogel – transferred to normoxia
**HLA-DR**	11639 ± 54	8694 ± 764	12563 ± 101	13362 ± 428
**CD40**	5985 ± 398	3416 ± 34	4671 ± 211	6065 ± 198
**CD86**	17342 ± 257	14237 ± 1122	18024 ± 644	19259 ± 998

### 3.5 O_2_-cryogels alleviate DC-mediated T-cell proliferation under hypoxic conditions

Mature DCs migrate to draining lymph nodes to initiate T-cell priming, triggering their activation and proliferation, thus inducing an adaptive immune response. However, low O_2_ levels impede T-cell proliferation and function, ultimately suppressing the activities of helper T cells and cytotoxic T cells (2, 48). Therefore, we explored the potential of O_2_-cryogels to preserve the allogeneic T-cell activation and proliferation, which is crucial for generating a robust adaptive immune response. First, LPS + IFN-γ-activated MDDCs were preconditioned in either normoxia or hypoxia using cryogel-free medium or medium containing various cryogels (O_2_-cryogel, HAGM cryogel) for 48 h. Subsequently, the activated MDDCs were co-cultured with CTV-labeled T cells from an independent donor, maintaining a 1:10 ratio, over a 6-day period in cryogel-free medium or medium containing various cryogels (O_2_-cryogel, HAGM cryogel). T cells stimulated with anti-CD3/anti-CD28 Dynabeads (aCD3/aCD28) and PMA-ionomycin were used as positive controls for the T-cell activation and proliferation studies (**Supplementary Figures 5A, B**). Next, the supernatants were collected to determine the levels of cytokines, and the cells were stained for T-cell lineage and activation markers. T-cell proliferation was assessed by monitoring CTV dilution using flow cytometry (**Figure 4**). T-cell proliferation was significantly reduced in hypoxia compared to normoxia (1.3 ± 0.4% *vs.* 34 ± 8.3%) (**Figures 4B–F**, **Supplementary Figures 5A, B**). Notably, O_2_-cryogels preserved T-cell proliferation in hypoxic conditions (10 ± 4%) by 30%. Similarly, hypoxic conditions significantly downregulated CD25, a T-cell activation marker, compared to normoxic conditions (5 ± 2% *vs.* 39 ± 9%), while O_2_-cryogels maintained CD25 levels by 50% (20 ± 6%) (**Figures 4G–K**). Hypoxic conditions also significantly suppressed the secretion of effector cytokines, such as Interferon gamma (IFN-γ), Interleukin-2 (IL-2), TNF-α, and TNF-β compared, to normoxia, which was prevented by O_2_-cryogel exposure (**Figures 4L–O**, **Table 3**). Strikingly, O_2_-cryogels preserved the secretion of IFN-γ by 30%, IL-2 by 57%, TNF-α by 76%, and TNF-β by 85%. Overall, our results suggest that O**_2_**-cryogels have the potential to maintain DC-mediated T-cell priming function in hypoxia, thereby partially retaining the activation and proliferation of T cells.

**Table 3:** Secretion of cytokines by DC-primed T cells.

Cytokines (pg/mL)	Normoxia	Hypoxia	Hypoxia + O_2_-cryogel
**IFN-γ**	16621 ± 3751	548 ± 178	5046 ± 1224
**IL-2**	816 ± 29	153 ± 32	461 ± 17
**TNF-α**	224 ± 48	21 ± 6	171 ± 61
**TNF-β**	1430 ± 152	368 ± 87	1219 ± 117

## 4 Discussion

Hypoxia within the tumor microenvironment (TME) is one of the major drivers of immunosuppression and is responsible for the limited efficacy of immunotherapy (53). Despite the encouraging outcomes of hypoxia-targeting therapies in both preclinical and clinical investigations, their effectiveness remains moderate and there exists a risk of systemic off-target toxicities (20–23). To overcome these challenges, we engineered a novel, O_2_-releasing biomaterial platform, designated O_2_-cryogels, and reported its ability to restore T cell-mediated cytotoxicity in a hypoxic and aggressive melanoma tumor model *in vitro* and *in vivo* (37–39, 54). Moreover, the process of biomaterial implantation is often associated with hypoxia due to lag in vascularization, thereby detrimental to immune cell viability and functions (41–45). In this regard, we have previously highlighted the co-adjuvant effect of O_2_ released from O_2_-cryogels in enhancing the efficacy of a protein-based COVID-19 vaccine (42). We demonstrated that mice immunized with an O_2_-cryogel-based COVID-19 vaccine exhibited a robust Th1 and Th2 immune response, resulting in the production of highly effective neutralizing antibodies against the SARS-CoV-2 virus. However, given the pivotal role of antigen-presenting cells, such as DCs, in initiating immune responses (40, 55), we leveraged O_2_-cryogel technology to investigate whether local oxygenation can preserve their activity under hypoxic conditions. In this context, the present study was designed to test the hypothesis that O_2_-cryogels can counteract the hypoxia-induced inhibition of human DC functions.

In this study, we designed O_2_-cryogels to sustain an O_2_ concentration above 1% in the cell culture media for 18–24 h, preventing MDDCs from encountering conditions below 1% O_2_ and thus avoiding deep hypoxic conditions. Indeed, we observed that O_2_-cryogels not only maintained the O_2_ tension in the cell culture media above 1% for 25 h but also extended the time it took for MDDCs to transition into a hypoxic state, from 3 h to 16 h. These findings suggest that O_2_-cryogels can modulate the microenvironment surrounding DCs, thereby influencing their function under adverse conditions. This is in line with previous studies showing that oxygenation can improve the function of immune cells (37, 42, 48). Even though the O_2_-release kinetics of O_2_-cryogels were sufficient for our *in vitro* studies, several strategies could be investigated to fine-tune the release kinetics *in vivo* for modulating immune cell responses. For instance, CaO_2_ particles can be coated with biodegradable polyesters such as poly(ϵ-caprolactone) (PCL) or poly (lactic-*co*-glycolic acid) (PLGA) to control and obtain a sustained release of O_2_. Additionally, alternate strategies to prolong O_2_ release can be explored, such as other oxides (e.g., zinc oxide, manganese dioxide) and peroxides (e.g., magnesium peroxide, H_2_O_2_), perfluorocarbons, and percarbonates (29, 41, 56–58).

Tumor hypoxia is known to hamper the antigen uptake function of DCs, thereby limiting their ability to phagocytose tumor antigens (12–14, 16). Herein, we initially demonstrated that O_2_-cryogels prevent hypoxia-induced suppression of antigen uptake by MDDCs. We showed that not only could O_2_-cryogels preserve up to 93% of the endocytic capacity of MDDCs under hypoxic conditions via O_2_ production but also HA plays an important role in salvaging the endocytic function of DCs. Notably, HA alone maintained 75% of the phagocytic activity of DCs under hypoxia. Therefore, our results highlight that HA-based O_2_-cryogels can be used to preserve the endocytic capacity of DCs to efficiently phagocytose pathogens and tumor antigens in hypoxic solid tumors. This activation, in turn, would initiate the innate immune cascade of antigen processing and presentation. However, additional studies are necessary to elucidate the mechanisms by which O_2_-cryogels modulate the antigen uptake function of DCs. For instance, their potential role in modulating mannose receptors, such as CD206 and CD209, which are involved in the endocytosis of pathogens, should be explored (51–54). Furthermore, chemotaxis is a crucial function of DCs, enabling their migration from inflamed tissues to lymph nodes, which are inherently hypoxic. This environment can alter their ability to present antigens to adaptive immune cells and mount a robust immune response (59–63). Interestingly, we have demonstrated that O_2_-cryogels could prevent hypoxia-induced inhibition of chemotaxis by MDDCs towards the CCL21 gradient, preserving 90% of the chemotactic activity under hypoxic conditions. Additionally, we have demonstrated the pivotal role of HA in enhancing the migratory ability of DCs, with HA alone contributing to 50% of the chemotactic activity of DCs in hypoxia. Collectively, our data strongly suggests that O_2_-cryogels can preserve the chemotactic capacity of DCs in hypoxia, enabling them to migrate to lymph nodes. This would ultimately enhance their capacity to initiate robust adaptive immune responses under hypoxic conditions. However, further investigations are required to understand the impact of O_2_-cryogels on the expression of matrix metalloproteinases in hypoxia. These enzymes, along with chemokine receptors, such as CCR7, are implicated in the migration of DCs from peripheral tissues to lymph nodes (18). Additionally, there is a need to dissect the mechanisms by which HA modulates DC functions. The interactions of HA with its receptors, including CD44, the receptor for hyaluronan-mediated motility (RHAMM/CD168), the HA receptor for endocytosis (HARE), and the lymphatic vessel marker LYVE-1, could potentially account for the HA-induced enhancement in antigen uptake and chemotactic activity of DCs (64–68). Furthermore, it is worth noting that different molecular weights of HA might have distinct impacts on DC functions (67, 69–71).

DCs undergo activation and maturation through various PRRs found on pathogens, such as LPS, and molecular patterns linked to viral infections, such as Poly (I:C) (52). Hypoxic conditions impact DC maturation, subsequently affecting their ability to initiate T-cell responses (16, 17). Moreover, DC morphology and size are indicative of their activation state, with immature DCs displaying a smaller size compared to matured DCs. Interestingly, our results show that O_2_-cryogels prevent the reduction in size of DCs by 90% in hypoxia, as depicted by their FSC properties, a parameter used to evaluate cell size by flow cytometry. While our microscopy images indicated no structural changes, differences in the cell density were observed between treatment groups. However, these differences did not translate to significant disparities in total cell number, as evaluated by flow cytometry. We attribute these observed differences in cell density to the technical aspects of our experiment, specifically the inadvertent removal of some cells along with the gels prior to imaging. Additionally, our findings demonstrate that O_2_-cryogels not only preserved their viability but also retained 80–100% of the levels of surface maturation and co-stimulation markers, such as HLA-DR, CD40, CD86, and CCR7, following stimulation with LPS and Poly (I:C). Notably, O_2_-cryogels boosted the levels of activation markers beyond those seen in normoxic levels, with an increase of 145% observed for CCR7, for instance. Moreover, O_2_-cryogels also preserved and enhanced the secretion profile of proinflammatory cytokines and chemokines such as IL-12p70, TNF-α, MCP-1, and MIP-1α (86–314%) by DCs under hypoxic conditions. Additionally, our results demonstrate that O_2_-cryogels can also restore the levels of activation markers on human DCs previously exposed to hypoxia. Overall, our results indicate that O_2_-cryogels can not only preserve but also reinstate the phenotypic maturation of human DCs, which is typically compromised within the TME. However, further research is necessary to elucidate the impact of the duration of hypoxia on the phenotype and functionality of DCs and the role of oxygenation in modulating these processes. Furthermore, it is crucial to conduct a comprehensive investigation into the differentiation and maturation processes of DCs under hypoxic conditions to replicate the complexities of the TME, which is often characterized by dynamic fluctuations in O_2_ levels and immunosuppressive factors. Additionally, it would be of significant interest to better understand how the introduction of O_2_ through O_2_-cryogels could influence this process.

Following activation and maturation, DCs migrate to the lymph nodes to prime T cells and stimulate their activation and proliferation. However, due to the inherent low O_2_ levels in the lymph nodes, T-cell activation and proliferation is suboptimal (2). Interestingly, O_2_-cryogels partially preserved T-cell activation by maintaining CD25 levels by 50% and conserved T-cell proliferation by 30% under hypoxic conditions. These findings suggest that O_2_-cryogels are capable of partially maintaining DC-mediated T-cell priming in hypoxia, thereby preserving T-cell activation and proliferation, which are pivotal for antitumor immunity. Even though DCs cultured under hypoxic conditions with O_2_-cryogels express CD86 and HLA-DR at levels similar to those cultured in normoxia, they only partially preserve DC-mediated T-cell activation and proliferation. This can be attributed to the 24-hour duration of O_2_ release by O_2_-cryogels in hypoxia. Given that our MLR setup spans a duration of 5–7 days, DCs and T cells will inevitably be exposed to hypoxia. This issue could potentially be addressed by extending the duration of O_2_ release, possibly through the use of perfluorocarbons or coating CaO_2_ particles with PCL) and PLGA. Additionally, further research may be necessary, potentially exploring O_2_-cryogels in combination with other immunomodulatory molecules to fully preserve the functions of T-cell priming, activation, and proliferation.

Furthermore, it is essential to delve into the inconsistencies arising from divergent outcomes in various studies investigating the effects of hypoxia on DCs (2, 3, 72). Even though there has been a consensus between the studies in terms of hypoxia inhibiting the antigen uptake by DCs (12–14), conflicting results have been observed in terms of hypoxia affecting the migratory capacity of DCs (13, 15, 16, 73). Additionally, inconsistent results were observed between different studies depicting the effects of hypoxia on maturation and expression of co-stimulatory markers on DCs upon LPS stimulation (12, 14–18, 74–81). Also, the impact of hypoxia on DC-mediated allogenic T-cell proliferation has been reported with inconsistent results (12, 13, 16, 77, 80, 82). These disparities could be attributed to various factors, such as varying the duration of hypoxia exposure, intermittent *vs.* continuous hypoxia, reoxygenation conditions, differentiation of DCs in hypoxia *vs.* normoxia followed by exposure to hypoxia, different experimental conditions, and the existing ambiguities regarding the actual O_2_ tension experienced by DCs under diverse experimental settings. Additionally, the lack of analyses on the phenotypes of DCs generated under different hypoxic experimental conditions could potentially contribute to the inconsistencies observed across various studies.

Moreover, further studies are needed to fully understand the mechanisms through which O_2_-cryogels enhance DC functions and to evaluate their potential *in vivo*. Possible mechanisms through which O_2_ generated by O_2_-cryogels can modulate DC functions include the degradation of HIFs, which are key regulators of cellular responses to low O_2_ conditions (83). Additionally, hypoxia can induce a shift in cellular metabolism from oxidative phosphorylation to glycolysis, subsequently influencing the differentiation, activation and apoptosis of DCs (84–86). Local oxygenation by O_2_-cryogels might prevent this metabolic shift, thereby preserving DC functions under hypoxic conditions. Hypoxia has also been reported to induce the expression of immune checkpoint molecules such as PD-L1 on DCs, leading to the inhibition of T-cell responses (19, 87, 88). The O_2_-cryogels might prevent the upregulation of these immune checkpoint molecules, through oxygenation, thereby preserving the ability of DCs to stimulate T-cell responses. While the current study did not specifically investigate DC-mediated antigen cross-presentation and CD8+ T-cell response under hypoxic conditions using O_2_-cryogels, we acknowledge the significance of this process, particularly in the context of tumor immunity. Future research will aim to explore this intriguing aspect using mouse and human DC cellular models, potentially providing further insights into the role of O_2_-cryogels in modulating immune responses.

In summary, our study provides strong evidence supporting the potential of O_2_-cryogels to preserve human DC functions under hypoxic conditions. Specifically, we have demonstrated that O_2_-cryogels can shield MDDCs from experiencing severe hypoxia, thereby preserving their antigen uptake, maturation, chemotaxis, and T-cell priming function. Additionally, we highlighted the crucial role of HA in shaping DC antigen uptake and chemotaxis function under hypoxic conditions. Given that approximately 80% of immunotherapies fail during clinical trials, most likely due to hypoxia-induced immunosuppression, the O_2_-cryogel platform holds substantial promise in unlocking the full potential of these therapies. Ultimately, this technology could pave the way for more clinically relevant and effective cancer treatments.

## Data availability statement

The original contributions presented in the study are included in the article/**Supplementary Material**. Further inquiries can be directed to the corresponding author.

## Ethics statement

Ethical approval was not required for the studies on humans in accordance with the local legislation and institutional requirements because only commercially available established cell lines were used.

## Author contributions

KB: Conceptualization, Investigation, Methodology, Validation, Writing – original draft, Writing – review & editing, Data curation, Formal analysis. AN: Investigation, Methodology, Writing – original draft. TC: Investigation, Methodology, Writing – original draft, Conceptualization, Data curation, Validation, Writing – review & editing. SB: Conceptualization, Investigation, Methodology, Validation, Writing – original draft, Writing – review & editing, Funding acquisition, Project administration, Resources, Supervision.

## References

[1] Liu K. Dendritic cells. In: Encyclopedia of Cell Biology. Academic Press (2016). p. 741–9. doi: 10.1016/B978-0-12-394447-4.30111-0

[2] Wang B Zhao Q Zhang Y Liu Z Zheng Z Liu S. Targeting hypoxia in the tumor microenvironment: a potential strategy to improve cancer immunotherapy. J Exp Clin Cancer Res (2021) 40:24. doi: 10.1186/s13046-020-01820-7 33422072 PMC7796640

[3] Winning S Fandrey J. Dendritic cells under hypoxia: how oxygen shortage affects the linkage between innate and adaptive immunity. J Immunol Res (2016) 2016:1–8. doi: 10.1155/2016/5134329 PMC4757696 26966693

[4] Muz B de la Puente P Azab F Azab AK. The role of hypoxia in cancer progression, angiogenesis, metastasis, and resistance to therapy. Hypoxia (2015) 83–92. doi: 10.2147/HP.S93413 PMC5045092 27774485

[5] Semenza GL Wang GL. A nuclear factor induced by hypoxia via *de novo* protein synthesis binds to the human erythropoietin gene enhancer at a site required for transcriptional activation. Mol Cell Biol (1992) 12:5447–54. doi: 10.1128/MCB.12.12.5447 PMC360482 1448077

[6] Kojima H Gu H Nomura S Caldwell CC Kobata T Carmeliet P. Abnormal B lymphocyte development and autoimmunity in hypoxia-inducible factor 1α-deficient chimeric mice. Proc Natl Acad Sci (2002) 99:2170–4. doi: 10.1073/pnas.052706699 PMC122337 11854513

[7] Thiel M Caldwell CC Kreth S Kuboki S Chen P Smith P. Targeted deletion of HIF-1α Gene in T cells prevents their inhibition in hypoxic inflamed tissues and improves septic mice survival. PloS One (2007) 2:e853. doi: 10.1371/journal.pone.0000853 17786224 PMC1959117

[8] Simko V Iuliano F Sevcikova A Labudova M Barathova M Radvak P. Hypoxia induces cancer-associated cAMP/PKA signalling through HIF-mediated transcriptional control of adenylyl cyclases VI and VII. Sci Rep (2017) 7:10121. doi: 10.1038/s41598-017-09549-8 28860539 PMC5578998

[9] Sitkovsky M Lukashev D. Regulation of immune cells by local-tissue oxygen tension: HIF1α and adenosine receptors. Nat Rev Immunol (2005) 5:712–21. doi: 10.1038/nri1685 16110315

[10] Sitkovsky MV Kjaergaard J Lukashev D Ohta A. Hypoxia-adenosinergic immunosuppression: tumor protection by T regulatory cells and cancerous tissue hypoxia. Clin Cancer Res (2008) 14:5947–52. doi: 10.1158/1078-0432.CCR-08-0229 18829471

[11] Harvey JB Phan LH Villarreal OE Bowser JL. CD73’s potential as an immunotherapy target in gastrointestinal cancers. Front Immunol (2020) 11:508. doi: 10.3389/fimmu.2020.00508 32351498 PMC7174602

[12] Yang M Ma C Liu S Sun J Shao Q Gao W. Hypoxia skews dendritic cells to a T helper type 2-stimulating phenotype and promotes tumour cell migration by dendritic cell-derived osteopontin. Immunology (2009) 128:e237–49. doi: 10.1111/j.1365-2567.2008.02954.x PMC2753916 19740309

[13] Ogino T Onishi H Suzuki H Morisaki T Tanaka M Katano M. Inclusive estimation of complex antigen presentation functions of monocyte-derived dendritic cells differentiated under normoxia and hypoxia conditions. Cancer Immunol Immunother (2012) 61:409–24. doi: 10.1007/s00262-011-1112-5 PMC11029581 21932134

[14] Elia AR Cappello P Puppo M Fraone T Vanni C Eva A. Human dendritic cells differentiated in hypoxia down-modulate antigen uptake and change their chemokine expression profile. J Leukoc Biol (2008) 84:1472–82. doi: 10.1189/jlb.0208082 18725395

[15] Qu X Yang M Kong B Qi L Lam QLK Yan S. Hypoxia inhibits the migratory capacity of human monocyte-derived dendritic cells. Immunol Cell Biol (2005) 83:668–73. doi: 10.1111/j.1440-1711.2005.01383.x 16266319

[16] Mancino A Schioppa T Larghi P Pasqualini F Nebuloni M Chen I-H. Divergent effects of hypoxia on dendritic cell functions. Blood (2008) 112:3723–34. doi: 10.1182/blood-2008-02-142091 18694997

[17] Bosseto MC Palma PVB Covas DT Giorgio S. Hypoxia modulates phenotype, inflammatory response, and leishmanial infection of human dendritic cells. APMIS (2010) 118:108–14. doi: 10.1111/j.1600-0463.2009.02568.x 20132174

[18] Zhao W Darmanin S Fu Q Chen J Cui H Wang J. Hypoxia suppresses the production of matrix metalloproteinases and the migration of humanmonocyte-derived dendritic cells. Eur J Immunol (2005) 35:3468–77. doi: 10.1002/eji.200526262 16259004

[19] Mortezaee K Majidpoor J Kharazinejad E. The impact of hypoxia on tumor-mediated bypassing anti-PD-(L)1 therapy. BioMed Pharmacother (2023) 162:114646. doi: 10.1016/J.BIOPHA.2023.114646 37011483

[20] Wigerup C Påhlman S Bexell D. Therapeutic targeting of hypoxia and hypoxia-inducible factors in cancer. Pharmacol Ther (2016) 164:152–69. doi: 10.1016/j.pharmthera.2016.04.009 27139518

[21] Hatfield SM Sitkovsky M. A2A adenosine receptor antagonists to weaken the hypoxia-HIF-1α driven immunosuppression and improve immunotherapies of cancer. Curr Opin Pharmacol (2016) 29:90–6. doi: 10.1016/j.coph.2016.06.009 PMC4992656 27429212

[22] Kjaergaard J Hatfield S Jones G Ohta A Sitkovsky M. A2A adenosine receptor gene deletion or synthetic A2A antagonist liberate tumor-reactive CD8+ T cells from tumor-induced immunosuppression. J Immunol (2018) 201:782–91. doi: 10.4049/jimmunol.1700850 PMC6052792 29802128

[23] Burroughs SK Kaluz S Wang D Wang K Van Meir EG Wang B. Hypoxia inducible factor pathway inhibitors as anticancer therapeutics. Future Med Chem (2013) 5:553–72. doi: 10.4155/fmc.13.17 PMC3871878 23573973

[24] Moen I Stuhr LEB. Hyperbaric oxygen therapy and cancer—a review. Target Oncol (2012) 7:233–42. doi: 10.1007/s11523-012-0233-x PMC3510426 23054400

[25] Leone RD Emens LA. Targeting adenosine for cancer immunotherapy. J Immunother Cancer (2018) 6:57. doi: 10.1186/s40425-018-0360-8 29914571 PMC6006764

[26] Roh M Wainwright DA Wu JD Wan Y Zhang B. Targeting CD73 to augment cancer immunotherapy. Curr Opin Pharmacol (2020) 53:66–76. doi: 10.1016/j.coph.2020.07.001 32777746 PMC7669683

[27] Gholipourmalekabadi M Zhao S Harrison BS Mozafari M Seifalian AM. Oxygen-generating biomaterials: A new, viable paradigm for tissue engineering? Trends Biotechnol (2016) 34:1010–21. doi: 10.1016/j.tibtech.2016.05.012 27325423

[28] Liang J-P Accolla RP Soundirarajan M Emerson A Coronel MM Stabler CL. Engineering a macroporous oxygen-generating scaffold for enhancing islet cell transplantation within an extrahepatic site. Acta Biomater (2021) 130:268–80. doi: 10.1016/j.actbio.2021.05.028 34087442

[29] Ashammakhi N Darabi MA Kehr NS Erdem A Hu S Dokmeci MR. Advances in controlled oxygen generating biomaterials for tissue engineering and regenerative therapy. Biomacromolecules (2020) 21:56–72. doi: 10.1021/acs.biomac.9b00546 31271024

[30] Sahu A Kwon I Tae G. Improving cancer therapy through the nanomaterials-assisted alleviation of hypoxia. Biomaterials (2020) 228:119578. doi: 10.1016/j.biomaterials.2019.119578 31678843

[31] Wang Z Chen T Li X Guo B Liu P Zhu Z. Oxygen-releasing biomaterials for regenerative medicine. J Mater Chem B (2023) 11:7300–20. doi: 10.1039/D3TB00670K 37427691

[32] Li X Wu Y Zhang R Bai W Ye T Wang S. Oxygen-based nanocarriers to modulate tumor hypoxia for ameliorated anti-tumor therapy: fabrications, properties, and future directions. Front Mol Biosci (2021) 8:683519. doi: 10.3389/fmolb.2021.683519 34277702 PMC8281198

[33] Accolla RP Liang J Lansberry TR Miravet IL Loaisiga M Sardi BL. Engineering modular, oxygen-generating microbeads for the *in situ* mitigation of cellular hypoxia. Adv Healthc Mater (2023) 12:2300239. doi: 10.1002/adhm.202300239 PMC10522802 36971050

[34] Zou M-Z Liu W-L Chen H-S Bai X-F Gao F Ye J-J. Advances in nanomaterials for treatment of hypoxic tumor. Natl Sci Rev (2021) 8(2):nwaa160. doi: 10.1093/nsr/nwaa160 34691571 PMC8288333

[35] Chen L Zheng B Xu Y Sun C Wu W Xie X. Nano hydrogel-based oxygen-releasing stem cell transplantation system for treating diabetic foot. J Nanobiotechnol (2023) 21:202. doi: 10.1186/s12951-023-01925-z PMC10294352 37370102

[36] Cheng F-Y Chan C-H Wang B-J Yeh Y-L Wang Y-J Chiu H-W. The oxygen-generating calcium peroxide-modified magnetic nanoparticles attenuate hypoxia-induced chemoresistance in triple-negative breast cancer. Cancers (Basel) (2021) 13:606. doi: 10.3390/cancers13040606 33546453 PMC7913619

[37] Colombani T Eggermont LJ Hatfield SM Rogers ZJ Rezaeeyazdi M Memic A. Oxygen-generating cryogels restore T cell mediated cytotoxicity in hypoxic tumors. Adv Funct Mater (2021) 31:2102234. doi: 10.1002/adfm.202102234 37745940 PMC10516343

[38] Bencherif SA Warren Sands R Ali OA Li WA Lewin SA Braschler TM. Injectable cryogel-based whole-cell cancer vaccines. Nat Commun (2015) 6:7556. doi: 10.1038/ncomms8556 26265369 PMC4763944

[39] Bencherif SA Sands RW Bhatta D Arany P Verbeke CS Edwards DA. Injectable preformed scaffolds with shape-memory properties. Proc Natl Acad Sci (2012) 109:19590–5. doi: 10.1073/pnas.1211516109 PMC3511752 23150549

[40] Bhatt K Eggermont LJ Bencherif SA. Polymeric scaffolds for antitumor immune cell priming. In: Engineering Technologies and Clinical Translation. Academic Press (2022). p. 63–95. doi: 10.1016/B978-0-323-90949-5.00003-6

[41] Pedraza E Coronel MM Fraker CA Ricordi C Stabler CL. Preventing hypoxia-induced cell death in beta cells and islets via hydrolytically activated, oxygen-generating biomaterials. Proc Natl Acad Sci (2012) 109:4245–50. doi: 10.1073/pnas.1113560109 PMC3306668 22371586

[42] Colombani T Eggermont LJ Rogers ZJ McKay LGA Avena LE Johnson RI. Biomaterials and oxygen join forces to shape the immune response and boost COVID-19 vaccines. Adv Sci (2021) 8:2100316. doi: 10.1002/advs.202100316 PMC8209904 34580619

[43] Suvarnapathaki S Wu X Lantigua D Nguyen MA Camci-Unal G. Breathing life into engineered tissues using oxygen-releasing biomaterials. NPG Asia Mater (2019) 11:65. doi: 10.1038/s41427-019-0166-2

[44] Guan Y Niu H Liu Z Dang Y Shen J Zayed M. Sustained oxygenation accelerates diabetic wound healing by promoting epithelialization and angiogenesis and decreasing inflammation. Sci Adv (2021) 7:eabj0153. doi: 10.1126/sciadv.abj0153 34452918 PMC8397271

[45] Niu H Li C Guan Y Dang Y Li X Fan Z. High oxygen preservation hydrogels to augment cell survival under hypoxic condition. Acta Biomater (2020) 105:56–67. doi: 10.1016/j.actbio.2020.01.017 31954189 PMC7098391

[46] Chometon TQ Siqueira M da S Santanna JC Almeida MR Gandini M Martins de Almeida Nogueira AC. A protocol for rapid monocyte isolation and generation of singular human monocyte-derived dendritic cells. PloS One (2020) 15:e0231132. doi: 10.1371/journal.pone.0231132 32271804 PMC7145147

[47] Rezaeeyazdi M Colombani T Memic A Bencherif S. Injectable hyaluronic acid-co-gelatin cryogels for tissue-engineering applications. Mater (Basel) (2018) 11:1374. doi: 10.3390/ma11081374 PMC6119876 30087295

[48] Colombani T Rogers ZJ Bhatt K Sinoimeri J Gerbereux L Hamrangsekachaee M. Hypoxia-inducing cryogels uncover key cancer-immune cell interactions in an oxygen-deficient tumor microenvironment. Bioact Mater (2023) 29:279–95. doi: 10.1016/j.bioactmat.2023.06.021 PMC10432785 37600932

[49] Rezaeeyazdi M Colombani T Eggermont LJ Bencherif SA. Engineering hyaluronic acid-based cryogels for CD44-mediated breast tumor reconstruction. Mater Today Bio (2022) 13:100207. doi: 10.1016/j.mtbio.2022.100207 PMC8844817 35198956

[50] Kim MK Kim J. Properties of immature and mature dendritic cells: phenotype, morphology, phagocytosis, and migration. RSC Adv (2019) 9:11230–8. doi: 10.1039/C9RA00818G PMC9063012 35520256

[51] Hong W Yang B He Q Wang J Weng Q. New insights of CCR7 signaling in dendritic cell migration and inflammatory diseases. Front Pharmacol (2022) 13:841687. doi: 10.3389/fphar.2022.841687 35281921 PMC8914285

[52] Kawai T Akira S. The role of pattern-recognition receptors in innate immunity: update on Toll-like receptors. Nat Immunol (2010) 11:373–84. doi: 10.1038/ni.1863 20404851

[53] Fu Z Mowday AM Smaill JB Hermans IF Patterson AV. Tumour hypoxia-mediated immunosuppression: mechanisms and therapeutic approaches to improve cancer immunotherapy. Cells (2021) 10:1006. doi: 10.3390/cells10051006 33923305 PMC8146304

[54] Rana D Colombani T Saleh B Mohammed HS Annabi N Bencherif SA. Engineering injectable, biocompatible, and highly elastic bioadhesive cryogels. Mater Today Bio (2023) 19:100572. doi: 10.1016/j.mtbio.2023.100572 PMC9984686 36880083

[55] Abdullah T Bhatt K Eggermont LJ O’Hare N Memic A Bencherif SA. Supramolecular self-assembled peptide-based vaccines: current state and future perspectives. Front Chem (2020) 8:598160. doi: 10.3389/fchem.2020.598160 33195107 PMC7662149

[56] Ícaro Sousa Morais A Wang X Vieira E Viana B Silva-Filho E Furtini J. Electrospraying oxygen-generating microparticles for tissue engineering applications. Int J Nanomed (2020) 15:1173–86. doi: 10.2147/IJN.S237334 PMC7037066 32110015

[57] Oh SH Ward CL Atala A Yoo JJ Harrison BS. Oxygen generating scaffolds for enhancing engineered tissue survival. Biomaterials (2009) 30:757–62. doi: 10.1016/j.biomaterials.2008.09.065 19019425

[58] Abdi SIH Ng SM Lim JO. An enzyme-modulated oxygen-producing micro-system for regenerative therapeutics. Int J Pharm (2011) 409:203–5. doi: 10.1016/j.ijpharm.2011.02.041 21356297

[59] Tiberio L Del Prete A Schioppa T Sozio F Bosisio D Sozzani S. Chemokine and chemotactic signals in dendritic cell migration. Cell Mol Immunol (2018) 15:346–52. doi: 10.1038/s41423-018-0005-3 PMC6052805 29563613

[60] Liu J Zhang X Cheng Y Cao X. Dendritic cell migration in inflammation and immunity. Cell Mol Immunol (2021) 18:2461–71. doi: 10.1038/s41423-021-00726-4 PMC8298985 34302064

[61] Zenewicz LA. Oxygen levels and immunological studies. Front Immunol (2017) 8:324. doi: 10.3389/fimmu.2017.00324 28377771 PMC5359232

[62] Wu H Estrella V Beatty M Abrahams D El-Kenawi A Russell S. T-cells produce acidic niches in lymph nodes to suppress their own effector functions. Nat Commun (2020) 11:4113. doi: 10.1038/s41467-020-17756-7 32807791 PMC7431837

[63] Ohta A Diwanji R Kini R Subramanian M Ohta A Sitkovsky M. *In vivo* T cell activation in lymphoid tissues is inhibited in the oxygen-poor microenvironment. Front Immunol (2011) 2:27. doi: 10.3389/fimmu.2011.00027 22566817 PMC3342240

[64] Lin C-A Ho H-M Venkatesan P Huang C-Y Cheng Y-J Lin Y-H. Hyaluronic acid-glycine-cholesterol conjugate-based nanoemulsion as a potent vaccine adjuvant for T cell-mediated immunity. Pharmaceutics (2021) 13:1569. doi: 10.3390/pharmaceutics13101569 34683862 PMC8539354

[65] Johnson LA Banerji S Lawrance W Gileadi U Prota G Holder KA. Dendritic cells enter lymph vessels by hyaluronan-mediated docking to the endothelial receptor LYVE-1. Nat Immunol (2017) 18:762–70. doi: 10.1038/ni.3750 28504698

[66] Johnson LA Jackson DG. Hyaluronan and its receptors: key mediators of immune cell entry and trafficking in the lymphatic system. Cells (2021) 10:2061. doi: 10.3390/cells10082061 34440831 PMC8393520

[67] Qhattal HSS Liu X. Characterization of CD44-mediated cancer cell uptake and intracellular distribution of hyaluronan-grafted liposomes. Mol Pharm (2011) 8:1233–46. doi: 10.1021/mp2000428 PMC3196641 21696190

[68] Toole BP. Hyaluronan-CD44 interactions in cancer: paradoxes and possibilities. Clin Cancer Res (2009) 15:7462–8. doi: 10.1158/1078-0432.CCR-09-0479 PMC2796593 20008845

[69] Moon S Shin E-C Noh Y-W Lim YT. Evaluation of hyaluronic acid-based combination adjuvant containing monophosphoryl lipid A and aluminum salt for hepatitis B vaccine. Vaccine (2015) 33:4762–9. doi: 10.1016/j.vaccine.2015.08.006 26271830

[70] Tolg C Hamilton SR Nakrieko K-A Kooshesh F Walton P McCarthy JB. Rhamm–/– fibroblasts are defective in CD44-mediated ERK1,2 motogenic signaling, leading to defective skin wound repair. J Cell Biol (2006) 175:1017–28. doi: 10.1083/jcb.200511027 PMC2064710 17158951

[71] Do Y Nagarkatti PS Nagarkatti M. Role of CD44 and hyaluronic acid (HA) in activation of alloreactive and antigen-specific T cells by bone marrow-derived dendritic cells. J Immunother (2004) 27:1–12. doi: 10.1097/00002371-200401000-00001 14676629

[72] Paardekooper LM Vos W van den Bogaart G. Oxygen in the tumor microenvironment: effects on dendritic cell function. Oncotarget (2019) 10:883–96. doi: 10.18632/oncotarget.26608 PMC6368231 30783517

[73] Filippi I Morena E Aldinucci C Carraro F Sozzani S Naldini A. Short-term hypoxia enhances the migratory capability of dendritic cell through HIF-1α and PI3K/akt pathway. J Cell Physiol (2014) 229:2067–76. doi: 10.1002/jcp.24666 24818793

[74] Pierobon D Bosco MC Blengio F Raggi F Eva A Filippi M. Chronic hypoxia reprograms human immature dendritic cells by inducing a proinflammatory phenotype and TREM-1 expression. Eur J Immunol (2013) 43:949–66. doi: 10.1002/eji.201242709 23436478

[75] Bosco MC Pierobon D Blengio F Raggi F Vanni C Gattorno M. Hypoxia modulates the gene expression profile of immunoregulatory receptors in human mature dendritic cells: identification of TREM-1 as a novel hypoxic marker in *vitro* and in *vivo* . Blood (2011) 117:2625–39. doi: 10.1182/blood-2010-06-292136 21148811

[76] Blengio F Raggi F Pierobon D Cappello P Eva A Giovarelli M. The hypoxic environment reprograms the cytokine/chemokine expression profile of human mature dendritic cells. Immunobiology (2013) 218:76–89. doi: 10.1016/j.imbio.2012.02.002 22465745

[77] Rama I Bruene B Torras J Koehl R Cruzado JM Bestard O. Hypoxia stimulus: An adaptive immune response during dendritic cell maturation. Kidney Int (2008) 73:816–25. doi: 10.1038/sj.ki.5002792 18216782

[78] Ricciardi A Elia AR Cappello P Puppo M Vanni C Fardin P. Transcriptome of hypoxic immature dendritic cells: modulation of chemokine/receptor expression. Mol Cancer Res (2008) 6:175–85. doi: 10.1158/1541-7786.MCR-07-0391 18314479

[79] Spirig R Djafarzadeh S Regueira T Shaw SG von Garnier C Takala J. Effects of TLR agonists on the hypoxia-regulated transcription factor HIF-1α and dendritic cell maturation under normoxic conditions. PloS One (2010) 5:e10983. doi: 10.1371/journal.pone.0010983 20539755 PMC2881864

[80] Lloberas N Rama I Llaudó I Torras J Cerezo G Cassis L. Dendritic cells phenotype fitting under hypoxia or lipopolysaccharide; adenosine 5′-triphosphate-binding cassette transporters far beyond an efflux pump. Clin Exp Immunol (2013) 172:444–54. doi: 10.1111/cei.12067 PMC3646444 23600833

[81] Paardekooper LM Bendix MB Ottria A de Haer LW ter Beest M Radstake TRDJ. Hypoxia potentiates monocyte-derived dendritic cells for release of tumor necrosis factor α via MAP3K8. Biosci Rep (2018) 38(6). doi: 10.1042/BSR20182019 PMC6294625 30463908

[82] Yang M Liu Y Ren G Shao Q Gao W Sun J. Increased expression of surface CD44 in hypoxia-DCs skews helper T cells toward a Th2 polarization. Sci Rep (2015) 5:13674. doi: 10.1038/srep13674 26323509 PMC4555176

[83] Luo Z Tian M Yang G Tan Q Chen Y Li G. Hypoxia signaling in human health and diseases: implications and prospects for therapeutics. Signal Transduct Target Ther (2022) 7:218. doi: 10.1038/s41392-022-01080-1 35798726 PMC9261907

[84] Al Tameemi W Dale TP Al-Jumaily RMK Forsyth NR. Hypoxia-modified cancer cell metabolism. Front Cell Dev Biol (2019) 7:4. doi: 10.3389/fcell.2019.00004 30761299 PMC6362613

[85] Kierans SJ Taylor CT. Regulation of glycolysis by the hypoxia-inducible factor (HIF): implications for cellular physiology. J Physiol (2021) 599:23–37. doi: 10.1113/JP280572 33006160

[86] Krzywinska E Stockmann C. Hypoxia, metabolism and immune cell function. Biomedicines (2018) 6:56. doi: 10.3390/biomedicines6020056 29762526 PMC6027519

[87] Song X Zhang Y Zhang L Song W Shi L. Hypoxia enhances indoleamine 2,3-dioxygenase production in dendritic cells. Oncotarget (2018) 9:11572–80. doi: 10.18632/oncotarget.24098 PMC5837754 29545920

[88] Noman MZ Desantis G Janji B Hasmim M Karray S Dessen P. PD-L1 is a novel direct target of HIF-1α, and its blockade under hypoxia enhanced MDSC-mediated T cell activation. J Exp Med (2014) 211:781–90. doi: 10.1084/jem.20131916 PMC4010891 24778419

