# Supplementary material for: Biomaterial-assisted local oxygenation safeguards the prostimulatory phenotype and functions of human dendritic cells in hypoxia
Source: Front Immunol. 2023 Dec 7;14:1278397. doi: 10.3389/fimmu.2023.1278397 (PMC10758617; doi:10.3389/fimmu.2023.1278397)
Supplement: Supplementary file 1 [file DataSheet_1.docx]

Supplementary Material

Biomaterial-assisted local oxygenation safeguards the prostimulatory phenotype and functions of human dendritic cells in hypoxia

Khushbu Bhatt^1^, Alexandra Nukovic^2^, Thibault Colombani^2^, Sidi A. Bencherif^2,3,4*^

^1^Department of Pharmaceutical Sciences, Northeastern University, Boston, MA, USA

^2^Department of Chemical Engineering, Northeastern University, Boston, MA, USA

^3^Department of Bioengineering, Northeastern University, Boston, MA, USA

^4^Harvard John A. Paulson School of Engineering and Applied Sciences, Harvard University, Cambridge, MA, USA

*** Correspondence:**Sidi A. Bencherif
s.bencherif@northeastern.edu


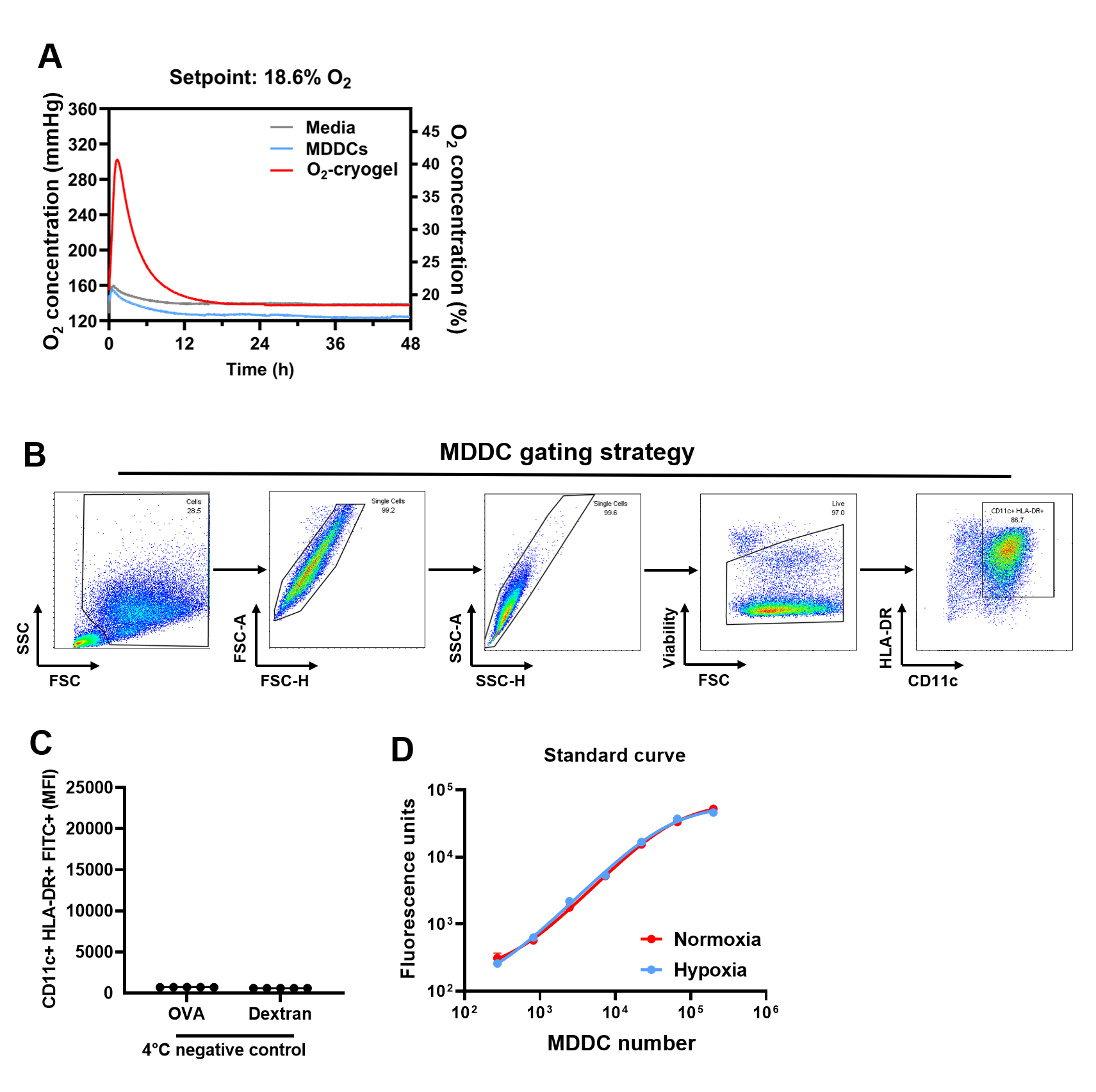
**Supplementary Figure 1. Characterization and analysis of O_2_ consumption, antigen uptake, and gating strategy of MDDCs.** (A) Profile of O_2_ consumption by MDDCs (1.5x10^5^ cells/well) and O_2_ release by O_2_-cryogels measured under normoxic conditions (18.6% O_2_) in cell culture media for 48 h using contactless sensor spots. Data are presented as mean of n = 4 replicates. (B) Flow cytometry gating strategy of MDDCs. Cells were gated out and debris was eliminated from forward and side scatter (FSC and SSC) properties. Following this, single cells were gated based on area (A) and height (H) parameters. Subsequently, live cells were selected, and then CD11c+ HLA-DR+ cells were gated, identifying them as MDDCs. (C) The uptake of OVA antigen and dextran by MDDCs at 4⁰C serves as a negative control for antigen uptake conditions at 37⁰C. Data are presented as mean ± SD of n = 4–5 replicates. (D) Standard curve generated using known number of serially diluted MDDCs in normoxic and hypoxic culture, followed by lysis using Lysis Buffer/Cyquant® GR Dye, and fluorescence units were quantified. Data are presented as mean ± SD of n = 2 replicates.


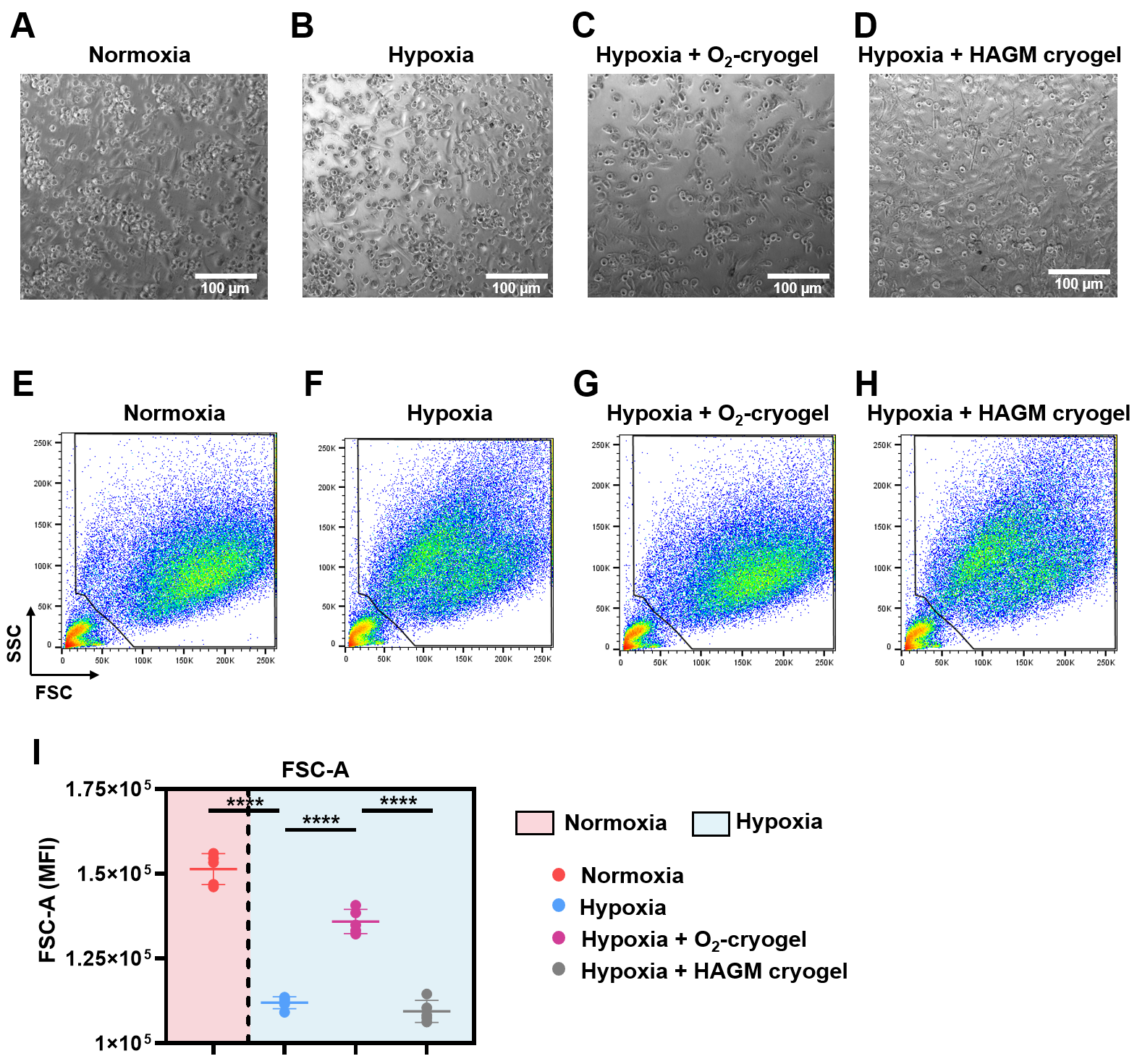


**Supplementary Figure 2.**  **O_2_-cryogels prevent activated MDDCs from contracting when exposed to hypoxia.** MDDCs were preconditioned in either normoxia or hypoxia in cryogel-free medium or medium containing various cryogels (O_2_-cryogel, HAGM cryogel) for 24 h in the presence of 2 µg/mL LPS and 50 ng/mL of IFN-γ. (A-D) Brightfield microscopy images of MDDCs under various experimental conditions. (E-I) Flow cytometry plots of SSC vs FSC of MDDCs stimulated with LPS and IFN-γ and quantification of FSC-A MFI under various experimental conditions. Data are representative of three independent experiments and presented as mean ± SD of n = 4–5 replicates. Statistical analysis was performed using one-way ANOVA and Tukey’s post hoc test using GraphPad software; *P < 0.05, **P < 0.01, ***P < 0.001, ****P < 0.0001.


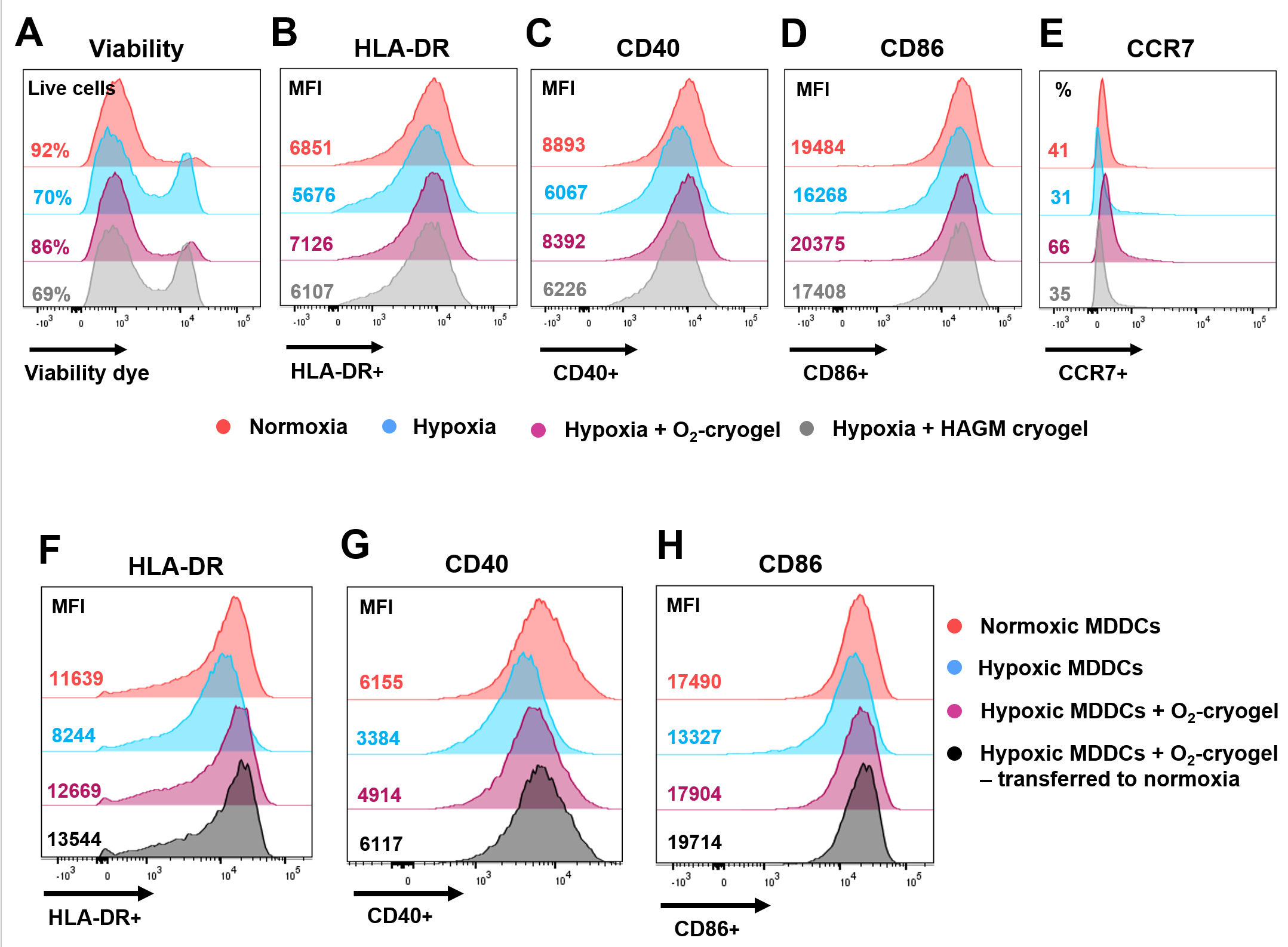


**Supplementary Figure 3. O_2_-cryogels safeguard the maturation of MDDCs in hypoxia.** (A-E) MDDCs were preconditioned in either normoxia or hypoxia in cryogel-free medium or medium containing various cryogels (O_2_-cryogel, HAGM cryogel) for 24 h in the presence of 2 µg/mL LPS and 50 ng/mL of IFN-γ. Histograms of (A) live MDDCs, (B) CD11c+ HLA-DR+, (C) CD11c+ HLA-DR+ CD40+, (D) CD11c+ HLA-DR+ CD86+ MDDCs, (E) Fraction of CD11c+ HLA-DR+ CCR7+ 24 h after culture under various experimental conditions. (F-H) MDDCs were stimulated with LPS (2 µg/mL) and IFN-γ (50 ng/mL) and subsequently subjected to different experimental conditions: (*i*) normoxia for 48 h, (*ii*) hypoxia for 48 h, (*iii*) preconditioned in hypoxia for 6 h, followed by addition of O_2_-cryogels and incubation in hypoxic conditions for another 42 h, and (*iv*) preconditioned in hypoxia for 6 h, followed by addition of O_2_-cryogels and incubation in hypoxic conditions for 18 h and subsequently transferred to normoxia for another 24 h. Histograms of (F) CD11c+ HLA-DR+, (G) CD11c+ HLA-DR+ CD40+, and (H) CD11c+ HLA-DR+ CD86+ MDDCs subjected to various experimental conditions. Data are representative of three independent experiments and presented as mean ± SD of n = 4–5 replicates. Statistical analysis was performed using one-way ANOVA and Tukey’s post hoc test using GraphPad software; *P < 0.05, **P < 0.01, ***P < 0.001, ****P < 0.0001.


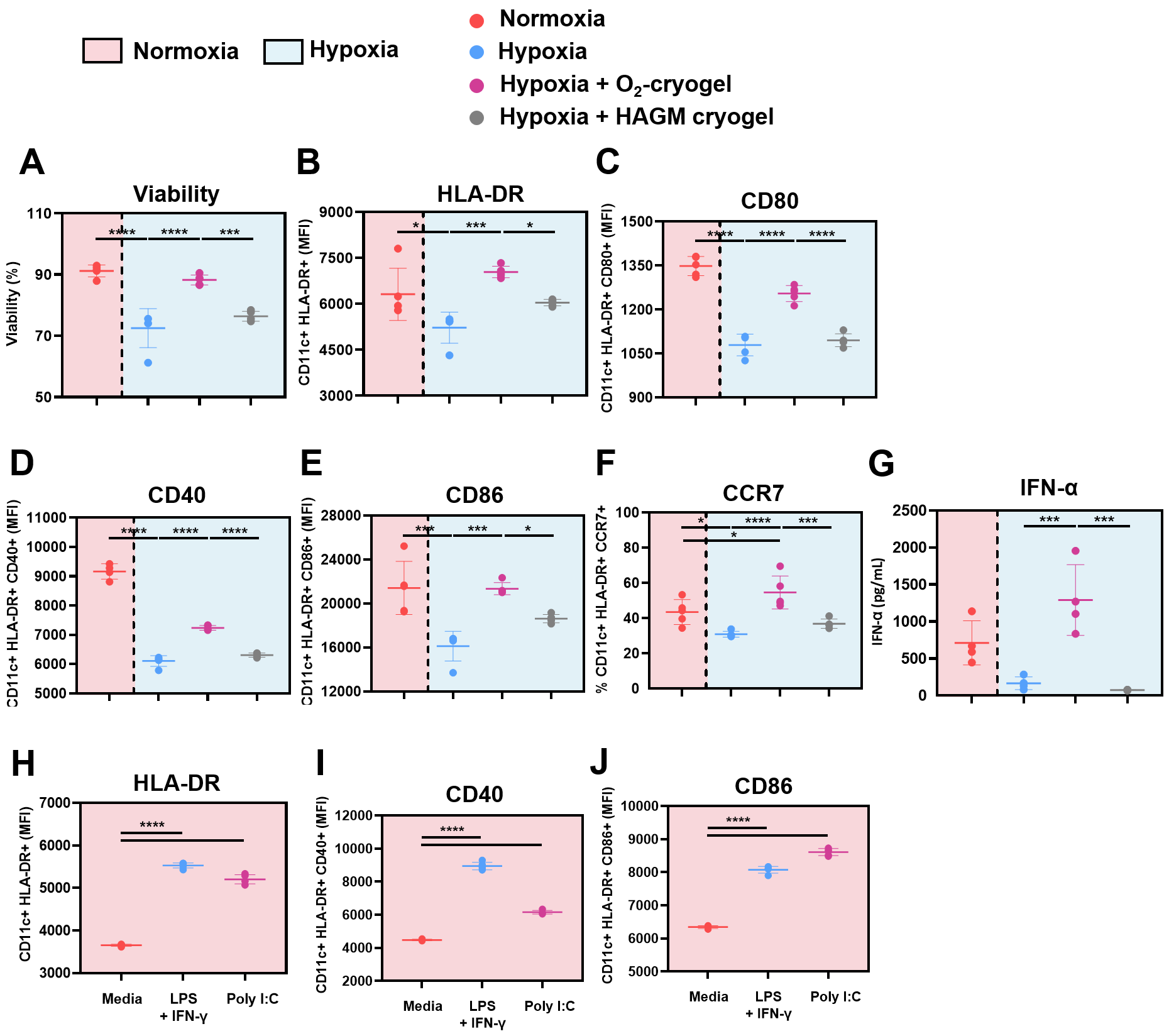


**Supplementary Figure 4. O_2_-cryogels mitigate hypoxia-induced inhibition of MDDC maturation.** MDDCs were preconditioned in either normoxia or hypoxia in cryogel-free medium or medium containing various cryogels (O_2_-cryogel, HAGM cryogel) for 24 h in the presence of 50 µg/mL Poly (I:C) (A-G) and 2 µg/mL LPS and 50 ng/mL of IFN-γ (H-J). (A) Fractions of live MDDCs, MFI of (B) CD11c+ HLA-DR+, (C) CD11c+ HLA-DR+ CD80+, (D) CD11c+ HLA-DR+ CD40+, (E) CD11c+ HLA-DR+ CD86+ MDDCs, (F) Fractions of CD11c+ HLA-DR+ CCR7+ MDDCs, and (G) secretion of IFN-α, 24 h after stimulation with Poly (I:C) under various experimental conditions. As a negative control for DC activation, LPS + IFN-γ and Poly (I:C) stimulation was compared with media only condition. MFI of (H) CD11c+ HLA-DR+, (I) CD11c+ HLA-DR+ CD40+, and (J) CD11c+ HLA-DR+ CD86+ 24 h after stimulation with media, LPS + IFN-γ and Poly (I:C) under normoxic conditions. Data are representative of three experiments and presented as mean ± SD of n = 4–5 replicates. Statistical analysis was performed using one-way ANOVA and Tukey’s post hoc test using GraphPad software; *P < 0.05, **P < 0.01, ***P < 0.001, ****P < 0.0001.


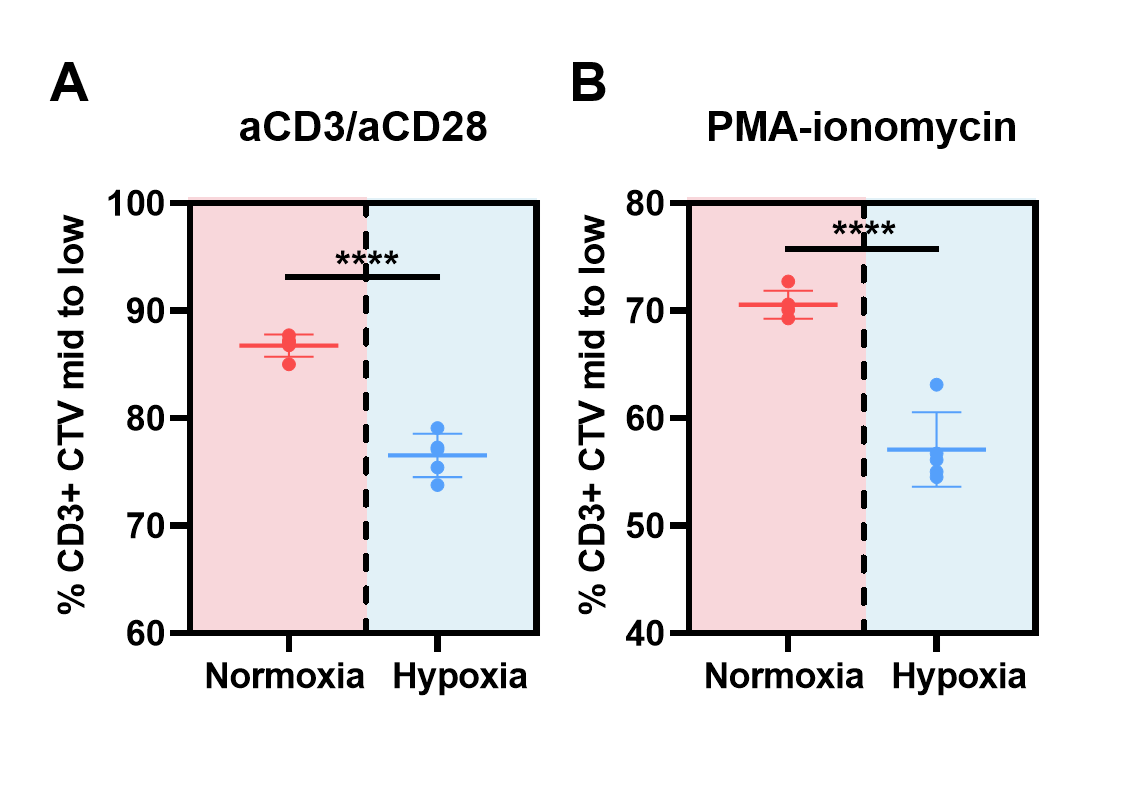


**Supplementary Figure 5. Hypoxia suppresses T-cell proliferation induced by anti-CD3/anti-CD28 dynabeads (aCD3/aCD28) and phorbol 12-myristate 13-acetate (PMA)-ionomycin.** CTV-labeled T cells were stimulated with aCD3/aCD28 dynabeads or PMA-ionomycin for 6 days as a positive control for mixed lymphocyte reaction. Subsequently, cells were stained for T-cell lineage and activation markers, and proliferation was assessed by monitoring CTV dilution using flow cytometry. Fractions of CD3+ CTV mid-low population upon (A) aCD3/aCD28 stimulation and (B) PMA-ionomycin stimulation. Data are representative of three experiments and presented as mean ± SD of n = 4–5 replicates. Statistical analysis was performed using one-way ANOVA and Tukey’s post hoc test using GraphPad software; *P < 0.05, **P < 0.01, ***P < 0.001, ****P < 0.0001.
